# Bioconjugation of Small Molecules to RNA Impedes Its Recognition by Toll-Like Receptor 7

**DOI:** 10.3389/fimmu.2017.00312

**Published:** 2017-03-24

**Authors:** Isabell Hellmuth, Isabel Freund, Janine Schlöder, Salifu Seidu-Larry, Kathrin Thüring, Kaouthar Slama, Jens Langhanki, Stefka Kaloyanova, Tatjana Eigenbrod, Matthias Krumb, Sandra Röhm, Kalina Peneva, Till Opatz, Helmut Jonuleit, Alexander H. Dalpke, Mark Helm

**Affiliations:** ^1^Institute of Pharmacy and Biochemistry, Johannes Gutenberg-University Mainz, Mainz, Germany; ^2^Department of Infectious Diseases, Medical Microbiology and Hygiene, University of Heidelberg, Heidelberg, Germany; ^3^Department of Dermatology, University Medical Center of the Johannes Gutenberg-University Mainz, Mainz, Germany; ^4^Institute of Organic Chemistry, Johannes Gutenberg-University Mainz, Mainz, Germany; ^5^Max Planck Institute for Polymer Research (MPG), Mainz, Germany

**Keywords:** bioconjugate, click chemistry, immunostimulation, mRNA, siRNA, small molecules, toll-like receptor

## Abstract

A fundamental mechanism of the innate immune system is the recognition, *via* extra- and intracellular pattern-recognition receptors, of pathogen-associated molecular patterns. A prominent example is represented by foreign nucleic acids, triggering the activation of several signaling pathways. Among these, the endosomal toll-like receptor 7 (TLR7) is known to be activated by single-stranded RNA (ssRNA), which can be specifically influenced through elements of sequence structure and posttranscriptional modifications. Furthermore, small molecules TLR7 agonists (smTLRa) are applied as boosting adjuvants in vaccination processes. In this context, covalent conjugations between adjuvant and vaccines have been reported to exhibit synergistic effects. Here, we describe a concept to chemically combine three therapeutic functions in one RNA bioconjugate. This consists in the simultaneous TLR7 stimulation by ssRNA and smTLRa as well as the therapeutic function of the RNA itself, e.g., as a vaccinating or knockdown agent. We have hence synthesized bioconjugates of mRNA and siRNA containing covalently attached smTLRa and tested their function in TLR7 stimulation. Strikingly, the bioconjugates displayed decreased rather than synergistically increased stimulation. The decrease was distinct from the antagonistic action of an siRNA bearing a Gm motive, as observed by direct comparison of the effects in the presence of otherwise stimulatory RNA. In summary, these investigations showed that TRL7 activation can be impeded by bioconjugation of small molecules to RNA.

## Introduction

Recognition of nucleic acids by the innate immune system results in the activation of signaling cascades that drive animal immune responses. Pattern-recognition receptors (PRRs) are tasked to discriminate between non-infectious self and potentially infectious non-self nucleic acids. This may be achieved by differences in structure, localization, and modification (1, 2). Recognition of non-self nucleic acids typically leads to an immune response that ultimately also shapes adaptive immunity. Precise definition of the structural details in nucleic acids that correspond to pathogen-associated molecular patterns (PAMPs) has important impact on our understanding of immune responses in bacterial and viral infections, autoimmune diseases, and cancer biology (2). Immediate impact of new insights will also affect the field of therapeutic nucleic acids (3, 4). Understanding the molecular details of innate nucleic acid recognition has made significant progress in the last couple of years with respect to cytosolic factors like retinoic acid inducible gene I (5–7), melanoma differentiation-associated protein 5 (8), absent in melanoma 2 (9–12), and more recently, cGAS (13, 14). Another class of membrane associated PRRs are toll-like receptors (TLRs), among which a subset is located to endosomes. These are thought to inspect exogenous material during the process of uptake and endocytosis. While TLR9 recognizes DNA; TLR3, TLR7/8, and murine TLR13 recognize microbial RNA. TLR3 recognizes double-stranded RNA above a minimal helix length of ~40 nucleotides (15), yet short siRNA might also induce activation in a different binding manner (16). TLR13 is activated by a 13-base sequence from bacterial 23S rRNA, and activation is sensitive to *N6*-methylation of a specific adenosine (17–20). PAMP recognition by the TLR7/8 system is particular in that RNA as well as a series of small molecules with structural elements from purine nucleobases are both recognized (21–26), albeit apparently associated to slightly different signaling modes (27, 28). Indeed, TLR7 and TLR8 were reported to bind degradation products of RNA at two different sites. A crystal structure of toll-like receptor 7 (TLR7) showed a presumed RNA degradation product, namely, guanosine (G), bound to a region that overlaps with a small molecules TLR7 agonists (smTLRa) binding site. Similarly, uridine was found in a TLR8 structure. Furthermore, a single-stranded RNA (ssRNA) was found binding to a distinct second binding site (28, 29). From these structures came the inspiration for a bioconjugate molecule offering ligands that might bind in both of the above binding sites. Whereas both TLR7 and TLR8 recognize RNA, their expression patterns in leukocytes differ (30). TLR7 is highly expressed in plasmacytoid dendritic cells (pDCs), which secrete type I IFN. Of note, pDCs are very nearly the exclusive contributors to IFN secretion from PBMCs, which is why PBMC preparations are popular in measurements of TLR7 stimulation *via* ELISA-based quantification of IFN in the supernatant after exposure of PBMCs to stimulating agents. In contrast, TLR8 is found in monocytes where stimulation induces TNF (31). While RNA recognition of this system has long been described as specific for ssRNA (2), recent results suggest that this simplified review is in need for some refinement. The recognition of mRNA (32) may still be attributed to its single-stranded regions, but tRNA contains very few truly single-stranded regions. Recognition of tRNA was evidenced in three domains of its structure, only one of which is truly single stranded (33, 34). These studies have also unraveled a particular mode of action of posttranscriptional modifications in the discrimination of self and non-self RNA. Ribose methylations in a specific sequence context (35) where shown to act as TLR7 antagonists (36), which do not only prevent the modified RNA from being sensed by TLR7 but also dampen response to additional unmodified, otherwise stimulatory RNA. Such modulation of TLR7 activation is of high interest in the design and development of therapeutic RNA, e.g., siRNA for diverse RNAi approaches (37, 38) or mRNA for tumor vaccine (39). In some approaches, an inhibition of TLR7 response is desirable, e.g., limiting immunostimulatory side effects by siRNA (40–42). In contrast, nucleic acid-derived adjuvants are frequently used to deliberately induce a boost of innate immune response, which, in turn, is known to increase the efficiency of certain vaccines (4, 43, 44). Ideally, it would be possible to fine-tune stimulatory properties *via* the nature and density of synthetic modifications on a therapeutic RNA. As a step in this direction, we decided to test, if the aforementioned TLR7 stimulation by mRNA and smTLRa could be further modulated by covalent conjugation to form a bidentate ligand reaching both binding sites of the receptor. Successful stimulation of innate immunity has been reported for covalent conjugates of various TLR ligands. In particular, ligands for TLR4, TLR7, and TLR9 have been combined by covalent conjugation in a single molecular entity and used to stimulate secretion of NFκB, IL-12, and other cytokines from bone-marrow derived DCs (45). Small molecule TLR7/8 agonists have been conjugated to various polymeric carriers thereby retaining their stimulatory properties. For example, the adenine derivative 1V270 was conjugated to a phospholipid *via* its *N*9 on the purine ring (46). *Via* the same site, another adenine derivative 1V209 was attached to polysaccharides (47). The same nitrogen, numbered *N*1 in tricyclic derivatives of the -quimod series (numbered I or 1 in Figure S1 in Supplementary Material), was used for conjugation of an imiquimod derivative to nanogels (48). In a similar concept, *N*1-derivatives of resiquimod (R848) to alkane and PEG chains leading to self-assembly of the compounds in to nanosized particles (49). Further, derivatives of the same compound class explored the *C*8 position (VIII in Figure S1 in Supplementary Material), the *C*2 position (II in Figure S1 in Supplementary Material), and exocyclic *N*4 (IV in Figure S1 in Supplementary Material), finding derivatization at these sites compatible with TLR7 stimulation (50, 51).

**Figure 1:**
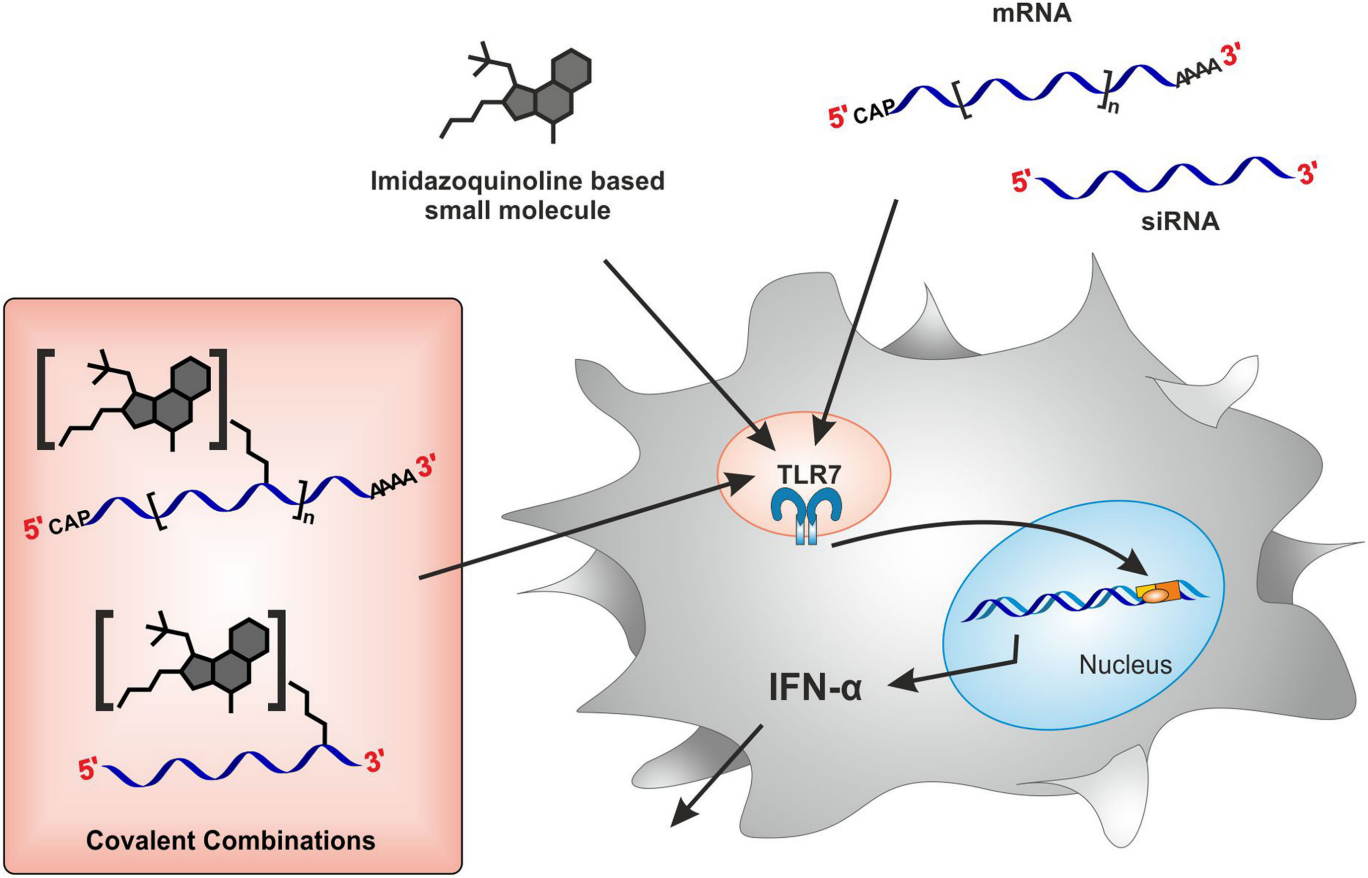
**Study conceptualization**. Stimulation of toll-like receptor 7 (TLR7) with either imidazoquinoline-based small molecules or RNA species such as mRNA and siRNA results in cytokine (IFNα) secretion from plasmacytoid DCs within PBMCs. How is TLR7 activity modulated upon covalent conjugation of both TLR7 ligands?

Based on the above findings, our concept, as depicted in Figure 1, aimed at the synthesis of a trifunctional mRNA, comprising two types of TLR agonists and the vaccine contained in the mRNA sequence itself. We chose the exocyclic *N*4 of resiquimod and the secondary amine in the *C*2-side chain of gardiquimod as attachment points for a bioconjugation approach that made use of click chemistry of the Cu(I)-catalyzed azide-alkyne 1,3-dipolar cycloaddition (CuAAC) type. Derivatization of this site, according to the recently published structure of TLR7 (28), is expected to disrupt only a single of the hydrogen bonds involved in the recognition of resiquimod, suggesting minimal interference with activity. The same structure suggested that a PEG chain might bridge the two identified binding sites in this receptor, one for resiquimod, and the other for RNA, potentially causing a cooperative effect from a bidentate ligand made of RNA and a small molecule derivative of resiquimod. Since RNAs bearing terminal alkyne groups are readily accessible, we synthesized azide derivatives with 1*H*-imidazo-[4,5-*c*]-purine structure (52–54), i.e., derivatives of imidazoquinolines of the quimod series and produced the respective bioconjugates for testing. While the anticipated cooperativity could not be evidenced, we observed that covalent modifications of RNA effectively decrease TLR7-mediated signaling.

## Materials and Methods

Details to the synthesis procedures of azide-modified TLR ligands, mannose- and dye-derivatives can be found in the supplementary section.

### Working with DNA and RNA

All DNA and RNA samples were handled in DNase/RNase- and endotoxin-free water (Zymo Research). Concentrations of DNA and RNA samples were determined using a NanoDrop™ spectrophotometer (Thermo Scientific). Additional confirmation of RNA concentration was carried out with a Qubit™ fluorometer (Thermo Scientific), excluding false positive results.

### pDNA Amplification and Preparation

For plasmid DNA, we used the transcription vector pGEM4Z64A-eGFP (55), which was transformed into competent DH5α *Escherichia coli* strain (Invitrogen) according to the manufacturer’s instructions and selected *via* an ampicillin resistance gene. pDNA was isolated from *E. coli* overnight culture following the Spin Format Protocol Modification of a GenElute™ high performance endotoxin-free plasmid maxiprep kit (Sigma-Aldrich). Plasmid linearization was carried out with the restriction enzyme *Bcu*I (Thermo Scientific) as described by the manufacturer, purified *via* phenol/chloroform extraction and followed by ethanol precipitation.

### mRNA Synthesis

mRNAs were transcribed *in vitro* from 5.0 μg linearized pDNA template using in house expressed and purified T7 RNA polymerase at 37°C for 4 h in a total volume of 100 μL Tris–HCl (40 mM, pH 8.1). Nucleoside triphosphates were applied in a 5 mM final concentration, whereas alkyne-modified 5-ethynyluridine-5′-triphosphate (EUTP) (Jena Bioscience, Germany) was used in indicated percentages of 5 mM and UTP in the remaining amount. Additionally, the reaction contained MgCl_2_ (30 mM), dithiothreitol (DTT 5 mM), spermidine (1 mM), and 0.01% Triton X-100. *In vitro* transcriptions (IVTs) were stopped by DNaseI treatment as described by the manufacturer (Thermo Scientific). Subsequent capping reactions were carried out using the combination of *Vaccinia* Capping System and mRNA Cap 2′-*O*-methyltransferase (NEB) following the one-step capping and 2′-*O*-methylation protocol (NEB) prolonged to 2 h. All *in vitro* transcripts and capped mRNA-constructs were purified using the MEGAclear™ Kit (Ambion™).

### Click Functionalization

All copper-catalyzed click reactions were performed in aqueous solutions containing up to 5% (v/v) dimethyl sulfoxide. The solutions were buffered to pH 8 with NaH_2_PO_4_ (100 mM) and contained 50 μg (5 μM) mRNA or 1 nmol sense siRNA (MH662; sequence see Supplementary Material, p. 26; IBA, Goettingen/Germany), respectively, 120–200 μM azido-functionalized ligand [synthesis and characterization for azide-compounds gardiquimod-diethylene-glycol-azide (GDA), resiquimod-polyethylene-glycol-azide (RPA), MMA, TMA, and PDI are given in the supplement]; SCy5-azide (Jena Bioscience, Germany), 250 μM CuSO_4_·5H_2_O, 1.25 mM *tris*-[4-(3-hydroxypropyl)-(1,2,3)triazolyl-1-methyl]amine, and 2.5 mM sodium ascorbate. The reaction mixtures were agitated under light protection at 25°C for 2 h. Reactions were stopped through addition of equivalent volumes of a 1 mM EDTA solution and purified through ethanol precipitation.

### Polyacrylamide Gel Electrophoresis (PAGE)

mRNA samples (1 μg) were dissolved in gel loading buffer [containing 20% glycerol in 1× TBE (Carl Roth^®^)] and loaded onto a 6% polyacrylamide gel. Electrophoresis was carried out in 1× TBE (Rotiphorese^®^, Carl Roth^®^) buffer at 12 W for 4 h. Gels were post-stained for 20 min with Stains-all (Sigma-Aldrich) and destained overnight in 75% isopropanol. Nucleic acid bands were visualized on a Typhoon 9400 (GE Healthcare) using 633 nm. Emission signals were recorded at 670 nm.

Single-stranded siRNA samples were analyzed by denaturing PAGE. Twenty-five picomoles of oligonucleotides were loaded onto a 20% denaturing polyacrylamide gel containing 1× TBE (compounds for denaturing PAGE from Carl Roth^®^). PAGE was performed in 1× TBE buffer (12 W/4 h), gels were then post-stained for 20 min with Stains-all (Sigma-Aldrich) and destained overnight in 75% isopropanol. Detection was carried out on a Typhoon 9400 (GE Healthcare), before and after staining, using 532 and 633 nm for excitation. Emission signals were recorded at settings 610BP30 nm and 670 nm.

### HPLC Analysis of EU-Containing mRNA

#### Sample Preparation

Prior to HPLC analysis, 20 pmol of each mRNA sample were digested to the nucleosides level according to the following protocol (56): samples were incubated in presence of 1/10 volume of 10× nuclease P1 buffer (0.2 M NH_4_OAc pH 5.0, ZnCl_2_ 0.2 mM), 0.3 U nuclease P1 (Sigma-Aldrich, Munich, Germany), and 0.1 U snake venom phosphodiesterase (Worthington, Lakewood, CO, USA) at 37°C for 2 h. Next, 1/10 volume of 10× fast alkaline phosphatase buffer (Fermentas, St. Leon-Roth, Germany) and 1 U fast alkaline phosphatase (Fermentas, St. Leon-Roth, Germany) were added, and samples were incubated for additional 60 min at 37°C. For the calibration series of EU, commercially available EU triphosphate was digested analogously.

#### HPLC Method

The digested mRNA samples were analyzed on an Agilent 1260 HPLC series equipped with a diode array detector (DAD). A Synergi Fusion-RP column (4 μm particle size, 80 Å pore size, 250 mm length, and 2 mm inner diameter) from Phenomenex (Aschaffenburg, Germany) was used at 35°C column temperature for the chromatographic separation of the nucleosides. The solvents applied were a 5 mM ammonium acetate buffer adjusted to pH 5.3 using acetic acid (solvent A) and pure acetonitrile (solvent B). The elution was performed at a flow rate of 0.35 mL/min using a linear gradient from 0 to 8% solvent B at 10 min, 40% solvent B at 20 min, and 0% solvent B at 23 min. For additional 7 min, the column was rinsed with 100% solvent A to restore the initial conditions. The detection of EU and the four canonical nucleosides was performed by measuring the column effluent photometrically at 254 nm using the DAD. For analysis of the recorded UV chromatograms and extracting the respective peak areas of EU and G, the Agilent MassHunter Qualitative Analysis software was used. The exact retention times of EU and the main nucleosides were determined using commercially available standard substances.

#### Quantification of EU in mRNA by HPLC Analysis

For quantification of EU in the mRNA samples, external calibration series were run for both EU (calibration range 2–120 pmol) and the G (calibration range 50–3,500 pmol) using commercially available reference substances. The detected peak areas for each calibration solution were plotted against the injected amount of EU or the G, and the slope of the linear fit of the resulting curves was used for calculation of the EU and G amounts in each sample. The amount of G was divided by the number of its sites per mRNA molecule, yielding the injected amount of mRNA molecules. The result was then used to calculate the amount of EU residues per mRNA (mol EU per mol mRNA).

### Stimulation of PBMCs

Human PBMCs were isolated from blood from voluntary healthy donors: informed consent was signed by each donor, and blood drawing was approved by the Ethic Committee of the Medical Faculty of the University Heidelberg (Permit S-157/2006). Heparinized blood was submitted to standard Ficoll-Hypaque density gradient centrifugation (Ficoll 1.078 g/mL) (42). PBMCs were resuspended in complete medium prepared of RPMI 1640 (Biochrom, Berlin, Germany) supplemented with 10% heat inactivated (1 h, 56°C) FCS (Gibco/Thermo Fisher Scientific, Schwerte, Germany). For stimulation, mRNA was encapsulated with DOTAP (*N*-[1-(2, 3-dioleoyloxy)propyl]-*N*,*N*,*N*-trimethylammonium-205 methylsulfate) (Carl Roth, GmbH Karlsruhe, Germany) at a ratio of 3 μL DOTAP per 1 μg of RNA in Opti-MEM Reduced Serummedium (Life Technologies) and incubation for 10 min at room temperature. As a control, cells were incubated with the individual clickable small molecule-, dye-, and mannose-derivatives only at indicated concentrations. Additionally, cells were co-stimulated with unmodified mRNA in the presence or absence of small molecules and their respective clickable derivatives. All stimulations were performed in duplicates per individual donor at a density of 4 × 10^5^ cells/well PBMCs in a 96-well flat bottom plate. Cells were incubated in a humidified 5% CO_2_ atmosphere at 37°C for 16–20 h. Cell-free supernatants were analyzed by sandwich ELISA for secretion of IFN-α (Affymetrix eBioscience, Frankfurt, Germany) according to the manufacturer’s protocol. Cytokines were detected by measuring the absorbance at 490 nm with a 650 nm reference in a photometer (Sunrise reader, Tecan, Salzburg, Austria). Cytokine concentration was calculated according to a standard dilution of recombinant cytokine using Magellan V 5.0 software (Tecan, Salzburg, Austria). Each experiment was repeated minimum three times. Cytokine secretion of individual donors was normalized to a stimulation with 1 μg/mL eGFP-mRNA or R848, respectively, which served as internal calibrator. Cell viability of stimulated PBMCs was assessed by MTS assay using CellTiter 96 Aqueous One solution proliferation kit (Promega, Madison, WI, USA) according to the manufacturer’s instructions. Cells were incubated for 3 h at 37°C in a humidified, 5% CO_2_ atmosphere. Viable cells were detected by measuring the absorbance at 492 nm in a photometer (Sunrise reader, Tecan, Salzburg, Austria).

### Generation and Transfection of Human Dendritic Cells

Myeloid DCs were generated from buffy coats of healthy volunteers as described previously (57, 58). In brief, PBMCs were isolated by Ficoll density gradient centrifugation, and monocytes were isolated by plastic adherence and cultured in X-VIVO-15 (Lonza) supplemented with 1% heat-inactivated autologous plasma, 800 IU/mL GM-CSF (Leukine, Berlex), and 100 IU/mL IL-4 (CellGenix). Fresh media with GM-CSF (1,600 U/mL) and IL-4 (100 IU/mL) were given at day 2 and day 4. Immature DCs were harvested at day 6 and subsequently used for further electroporation experiments. All electroporation experiments with human DCs were performed with Neon Transfection System (Thermo Fisher Scientific). According to the manufacturer’s instruction, 0.5–1 × 10^6^ DCs were electroporated with various amounts of mRNA in a total volume of 100 μL of electroporation buffer. To achieve high transfection efficiencies, the following program was used: pulse voltage: 1,500 V; pulse width: 30 ms; pulse number: 1. Afterward, DCs were cultured in pre-warmed X-VIVO-15 supplemented with 1% heat-inactivated autologous plasma, 800 IU/mL GM-CSF and 100 IU/mL IL-4 for 24 h at 37°C, 5% CO_2_. RNA translation was analyzed by flow cytometry (BD Accuri™ C6 Cytometer).

### Knockdown in HeLa MAZ

#### Cells

HeLa MAZ cells (59) contain the episomal vector pMARS-mODC-AZ, which encodes for a destabilized eGFP. Cells were a kind gift from Dr. Andriy Khobta from the group of Prof. B. Epe (Institute of Pharmacy and Biochemistry, Mainz).

#### Hybridization

siRNA single strands (antisense MH533 and sense MH662; sequences see Supplementary Material) were obtained from IBA (Göttingen, Germany). The hybridization experiments were carried out in 1× phosphate-buffered saline (pH 7.4), with the two complementary strands in a 1:1 ratio, to result in a final duplex concentration of 5 μM. The strands were first incubated at 70°C for 3 min, and duplex formation was allowed at 37°C over 1 h. The prepared duplex siRNA was stored at −20°C.

#### Knockdown Experiments

Prior to transfection, 5 × 10^4^ HeLa MAZ cells were seeded in 24-well plate in 1 mL DMEM (Thermo Fisher) with 10% fetal bovine serum (Sigma-Aldrich). After 1 day, medium was replaced by 500 μL of 10% FCS DMEM, and cells were transfected with siRNA. Briefly, to prepare siRNA/lipid transfection mixture, 40 pmol from a starting 5 μM siRNA duplex was diluted in Opti-MEM^®^ (Thermo Fisher) in twofold dilution series and mixed with transfection agent Lipofectamine™ (Thermo Fisher) according to the manufacturer’s instruction. In the transfection time, 100 μL of siRNA was added in dropwise to the wells. Transfection experiment was realized in duplicate, and each experiment was repeated three times. Cells were incubated 24 h, after which the medium was replaced by 185 μL of 10% FCS medium and 65 μL of 2 M MG115 (proteasome inhibitor, Sigma-Aldrich). This was followed by another 6 h incubation. For FACS analysis, cells were washed with 500 μL DPBS, trypsinized with 200 μL trypsin/EDTA, resuspended in 400 μL DPBS, and the eGFP signal measured by flow cytometry instrument (LSR-FortessaSORP, BD Biosciences) with excitation at 488 nm and a 530BP30 nm emission filter. Data were used for IC_50_ curves. The calculated eGFP signal corresponds to the product of the percentage of eGFP positive cells and their median fluorescence intensity, normalized to the value of positive controls (untreated with siRNA duplex). For acquisition and analysis, the FACSDiva Software (BD Biosciences) was used.

### Statistical Analysis

Data were analyzed using GraphPad Prism 7.0 (GraphPad Software Inc.). Significant differences were assessed by two-way ANOVA followed by multiple comparisons tests. In all figures, the *P* values are indicated by ns (not significant; *P* > 0.05), *P ≤ 0.05, **P ≤ 0.01, ***P ≤ 0.001, ****P ≤ 0.0001.

## Results

The original question we sought to address, derived from the recent report of two distinct signaling pathways originating from TLR7 stimulation, one triggered by small molecules of the imiquimod series, and the other triggered by RNA (27, 28). We wondered, if it was possible to simultaneously stimulate both pathways by chemically combining both sorts of PAMPs in the same molecule. Hence, we designed small molecule derivatives of the -quimod series with two alternative sites for immobilization on RNA molecules by CuAAC-click chemistry. RNA molecules could then be viewed as scaffolds to present both types of TLR7-activating molecular patterns. To this end, we used alkyne-modified siRNA as well as alkyne-modified mRNA, thus a small and a large RNA, both considered for therapeutic purposes (4, 39, 60) and both reported to be TLR7 ligands (61, 62).

### Azide-Functionalized Small Molecule TLR Agonists: TLR7 Activity Depends on Conjugation Site

**Figure 2:**
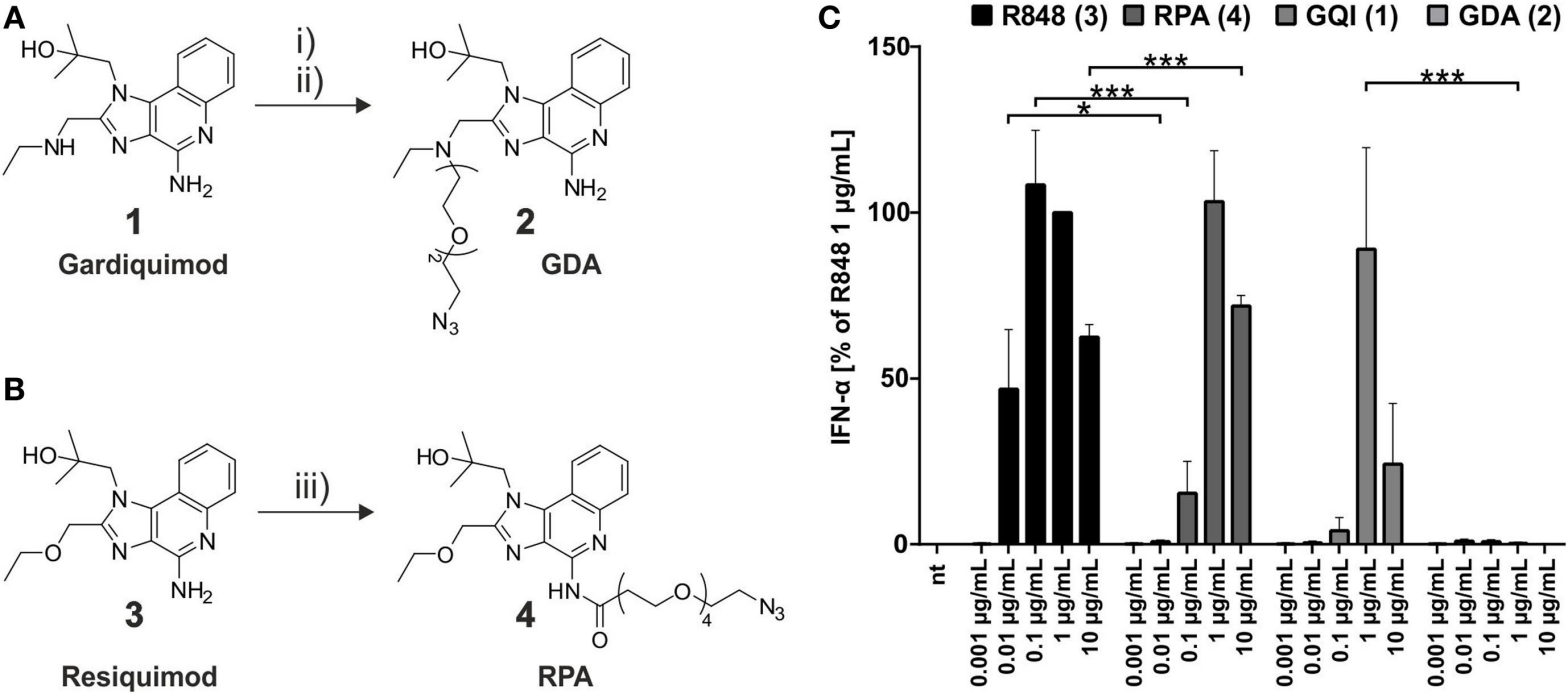
**Synthesis of azide-functionalized small molecule toll-like receptor agonists**. **(A)** gardiquimod-diethylene-glycol-azide (GDA) (2) from gardiquimod (1): (i) mesylation of HO-TEG-N_3_, 80%; (ii) SO_2_MeO-TEG-N_3_, acetonitrile, RT, 50%. **(B)** Resiquimod-polyethylene-glycol-azide (RPA) (resiquimod-PEG_4_-N_3_) (4) from resiquimod (R848) (3): (iii) NHS-PEG_4_-N_3_, DCM, RT, 25%. **(C)** Titration of PBMCs with the commercial small molecules and their respective azide derivatives (nt, non-treated). IFN-α production was measured by ELISA as technical duplicate of biological triplicates (three donors). Due to donor variation in the absolute amount of IFN-α secreted, data from each individual were normalized to 1.0 μg/mL R848 (=100%) of the respective (*n* = 3; mean + SD). [Asterisks above bars indicate the respective *P* values evaluated by ANOVA and Sidak’s multiple comparisons test; no declaration = not significant (ns).]

The synthesis route to azide-bearing small molecule TLR ligands is depicted in Figure 2 below. In order to equip gardiquimod **1** (GQI) with an azido-ethylene glycol linker at its aliphatic amine, the hydroxyl group of the latter was converted into a good leaving group, a methane sulfonyl moiety. This linker was attached to the small molecule *via* substitution at the exocyclic secondary amine to give the desired product GDA **2**. Resiquimod **3** (R848) was equipped with an azido-polyethylene-glycol linker by applying standard NHS-ester chemistry (63) between its primary amine and the reactive *N*-hydroxysuccinimide-group of the linker to yield the product RPA **4**. The purity of both products was confirmed by ^1^*H*-NMR, full range and high resolution MS (Figures S2–9 in Supplementary Material), which was a prerequisite for subsequent experiments, to exclude residual starting material of the smTLRa.

The impact on immunostimulatory activity arising from the conjugates in contrast to the original agonists was evaluated by an ELISA-based measurement of IFN-α secretion from incubated PBMCs, which reflects activation of pDCs through TLR7 (33, 64). As the highly significant comparison in Figure 2C (all results of significance evaluation given in Table S1 in Supplementary Material) shows attachment of an azide-conjugated PEG-linker at the *C*-2-ethyl-amino-methyl-group of the 1*H*-imidazo-[4,5-*c*]-quinolin scaffold (see also Figure S1 in Supplementary Material) ablated TLR7 stimulation of the gardiquimod derivative 2. This finding was in keeping with a previous study reporting diminished IFN secretion upon variation at the *C*-2 site (51), although others reported the *N*-9-position as a “tolerant” linker site upon structure–activity relationship measurements (52–54). In contrast, attachment of the PEG-linker to the *C-*4-NH_2_-group of 3, which resulted in the resiquimod derivative 4, led to less stimulation than 3 at a concentration of 0.1 μg/mL, but to an equal outcome at a concentration of 1 μg/mL and even higher at 10 μg/mL (see also Table S1 in Supplementary Material). Thus, the conjugation to a PEG chain, while it indeed did diminish the activity of 4, still allowed to retain activity that showed no difference up to a significant enhancement to unconjugated gardiquimod (Table S1 in Supplementary Material), which itself is a potent agent originally developed as a potential successor of imiquimod. The activity of 4 is in keeping with the conjugation chemistry interfering with receptor binding only at a single hydrogen bond (28). The linker-equipped resiquimod 4 is therefore a valid smTLRa for later comparison with its mRNA-conjugate. Of note, an MTS-based cell viability assay (Figure S15 in Supplementary Material) showed decreased metabolic activity after exposure to 10 μg/mL resiquimod, which likely explains the reduced IFN secretion under these conditions. However, cells showed normal viability under all other conditions.

### Synthesis of Alkyne-Modified mRNA and Posttranscriptional Functionalization

Using eGFP encoding mRNA as a model that allowed reporting its functionality in protein biosynthesis, we synthesized alkyne-modified mRNA by IVT with T7 RNA polymerase from a linearized plasmid-DNA template comprising a poly-dT sequence of 64 dTs for the *in situ* synthesis of a 3′-poly-A-tail. For alkyne-modified mRNAs, 1 or 10% of the standard UTP reaction concentration were substituted with EUTP (65, 66), with no discernible impact on the IVT yield. The 5′-end of the purified IVT-construct was subsequently equipped with a 7-methylguanosine-ppp-Gm cap structure (Cap1) (Figure S16A in Supplementary Material). This was effected by means of combined enzymatic reactions of the *vaccinia* capping enzyme and 2′-*O*-methyltransferase (67, 68) after optimization employing a tritium incorporation assay with ^3^H-*S*-adenosyl-methionine (Figure S16B in Supplementary Material).

**Figure 3:**
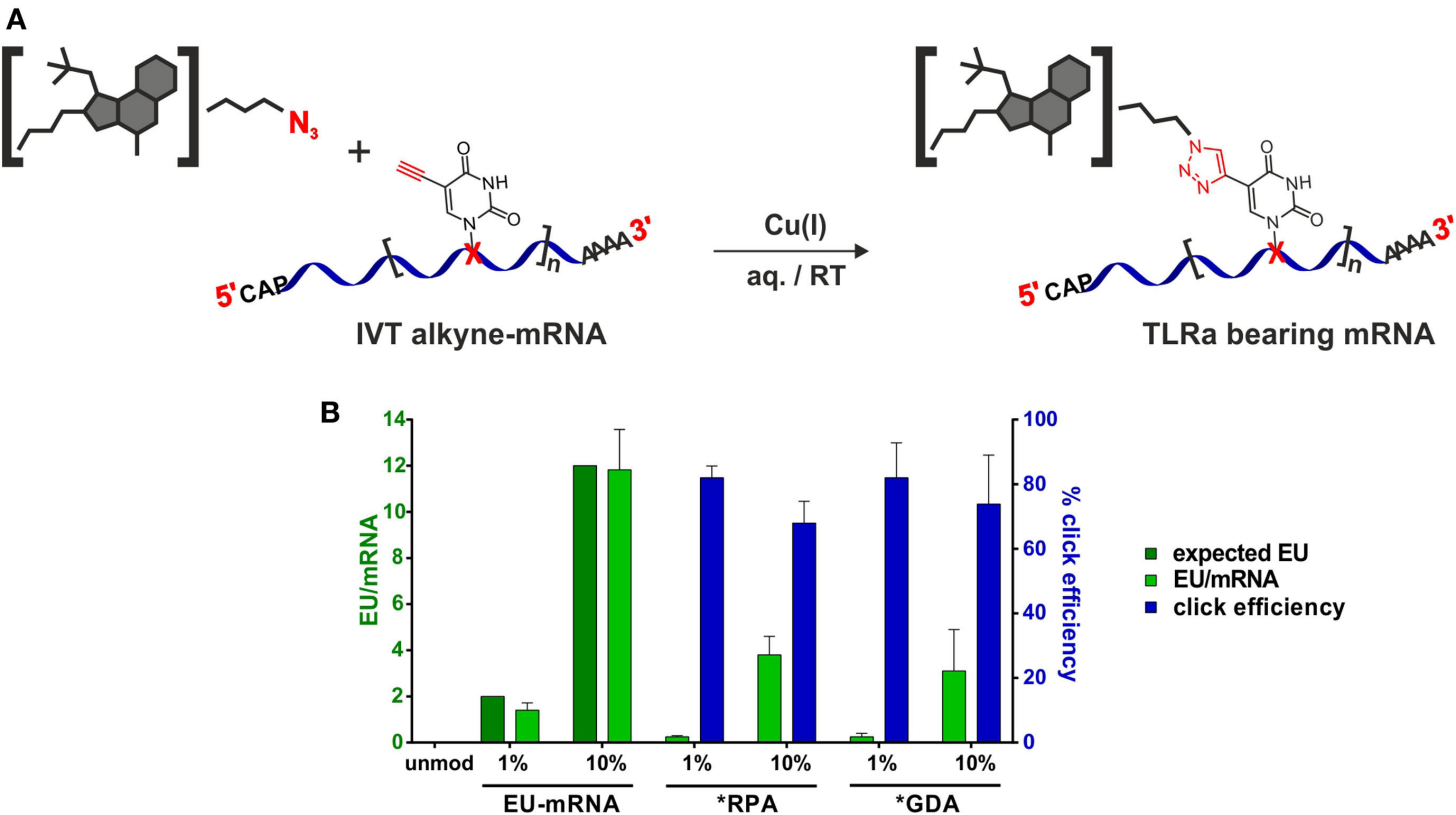
**Synthesis of small molecule-TLRa bearing eGFP-mRNA**. **(A)** Symbolic synthesis scheme of Cu(I)-catalyzed azide-alkyne-cycloaddition between azide-functionalized toll-like receptor (TLR) agonists and *in vitro* transcribed (n)alkyne-modified eGFP-mRNA. **(B)** HPLC-quantification of EU content (light green) in *in vitro* transcription (IVT)- and TLRa-eGFP-mRNA including click efficiency (blue) (*n* = 5; mean + SD).

With an mRNA equipped with terminal alkyne moieties and azide-functionalized small molecule derivatives (Figure 3A) in hand, CuAAC-click reactions were conducted according to Hong et al. (69). Integrity of the mRNA after click reaction was verified by PAGE (Figure S17 in Supplementary Material).

To assess incorporation of clickable nucleoside and click efficiency, both, alkyne-containing *in vitro* transcripts and products from click reactions, were analyzed on the nucleoside level with respect to their existing/remaining EU content. Therefore, the RNA oligonucleotides were digested to nucleosides by stepwise incubation with nuclease P1, snake venom phosphodiesterase, and alkaline phosphatase and subjected to quantitative HPLC analysis. With the sequence of the unmodified mRNA containing 119 uridines (Supplementary Material, p. 26), it was expected to find 1–2 and 12, respectively, of them replaced by 5-ethynyluridine in IVT syntheses when employing 1 and 10% EU, respectively. Figure 3B shows quantification results (light-green bars for EU-mRNA) confirming this assumption.

The yield of the implemented click reactions was determined from residual EU (light green and blue bars in Figure 3B). For both ligands, click modification of 1% EU-mRNA proceeded to 82% completion, corresponding to 1–2 conjugated small molecules per molecule mRNA, and to 70% of the 10% EU-mRNA, equaling 8–9 conjugated small molecules per molecule mRNA.

**Figure 4:**
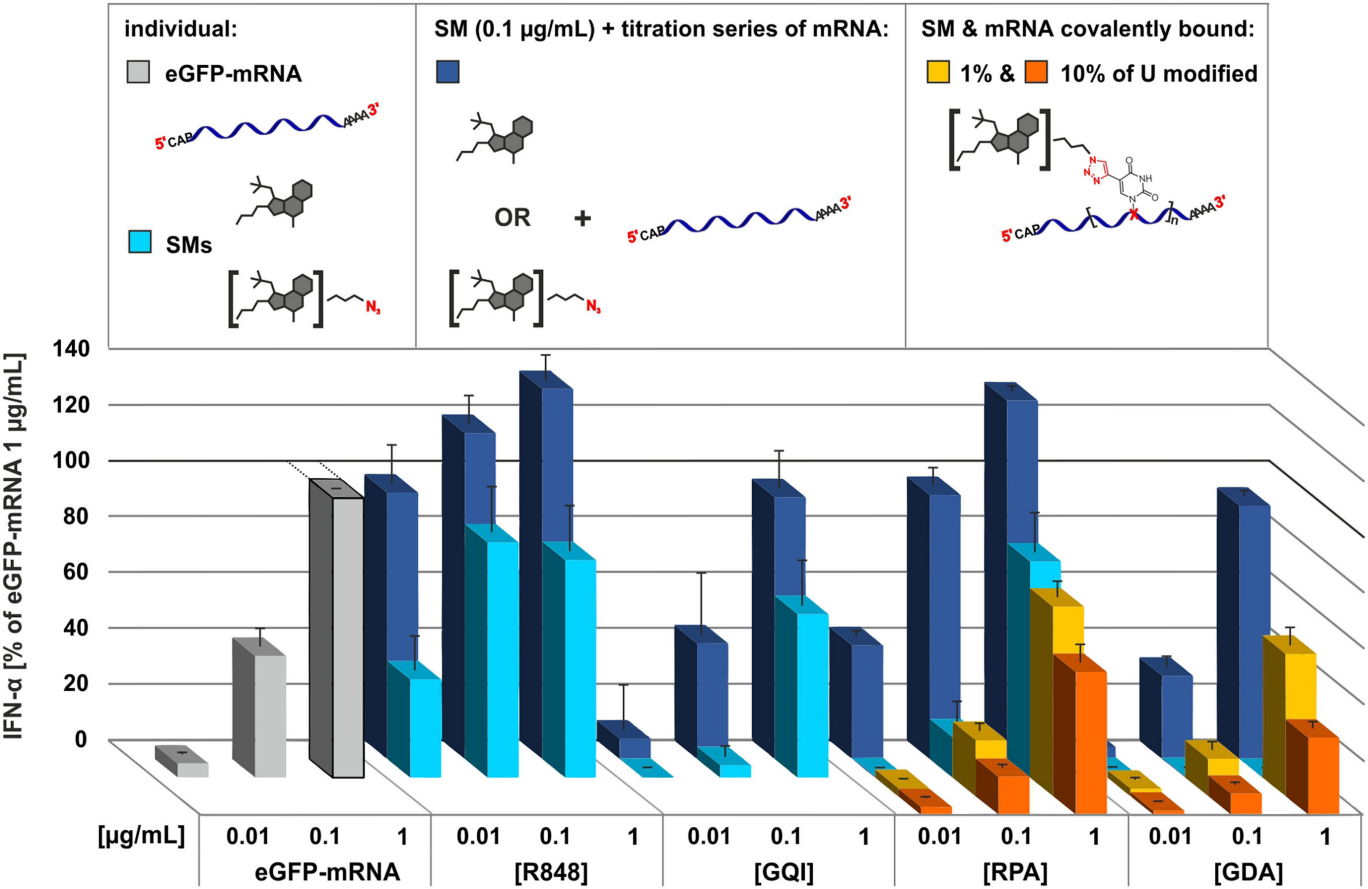
**Comparison of the effect of small molecules TLR7 agonists, RNA, and covalent conjugates of both in immunostimulation**. Titration of PBMCs with eGFP-mRNA (gray), commercial small molecules and their respective azide derivatives (light blue), 0.1 μg/mL of SMs titrated individually with eGFP-mRNA (dark blue), 1% (yellow) and 10% (orange) alkyne-eGFP-mRNA clicked with resiquimod-polyethylene-glycol-azide (RPA) and gardiquimod-diethylene-glycol-azide (GDA), respectively. IFN-α production was measured by ELISA as technical duplicate of biological triplicates (three donors). Due to donor variation in the absolute amount of IFN-α secreted, data from each individual were normalized to 1.0 μg/mL eGFP-mRNA (=100%) of the respective (*n* = 3; mean + SD). Numeric *P* values are given in Figure S18 in Supplementary Material.

To gauge the dynamic range of a potential cooperative stimulation by both types of TLR7 agonists, they were tested together. Therefore, unmodified mRNA concentration was varied at a constant concentration (0.1 μg/μL) of the smTLRa. Figure 4 shows that smTLRa based IFN-α secretion can be increased by addition of mRNA (dark blue bars). In particular, the maximum effect of resiquimod, determined to be at 0.1 μg/mL in Figure 2C, was increased as a function of the concentration of additional mRNA (Figure 4; Figure S18 in Supplementary Material). Similarly, IFN-α secretion based on RPA, gardiquimod, or GDA alone, was increased upon addition of mRNA.

According to the working hypothesis, a relative increase upon stimulation with the covalent conjugates was expected. As shown in Figure 4, the impact on covalent attachment of the TLRa derivatives to mRNA in terms of TLR7 stimulation contradicted this original hypothesis. Rather than showing an amplification or synergistic effect, the TLRa moieties clicked onto the mRNA (yellow and orange bars) dampen the emission of IFN-α in comparison to free mRNA (gray bars) or the combined mRNA and smTLRa (dark blue bars). The effect is mild at 1–2 TLRa moieties per molecule mRNA but clearly more pronounced at a higher degree of modification, i.e., 8–9 moieties per mRNA.

### Non-TLR-Binding Moieties also Shield RNA-Conjugate Molecules from Stimulating TLR7

**Figure 5:**
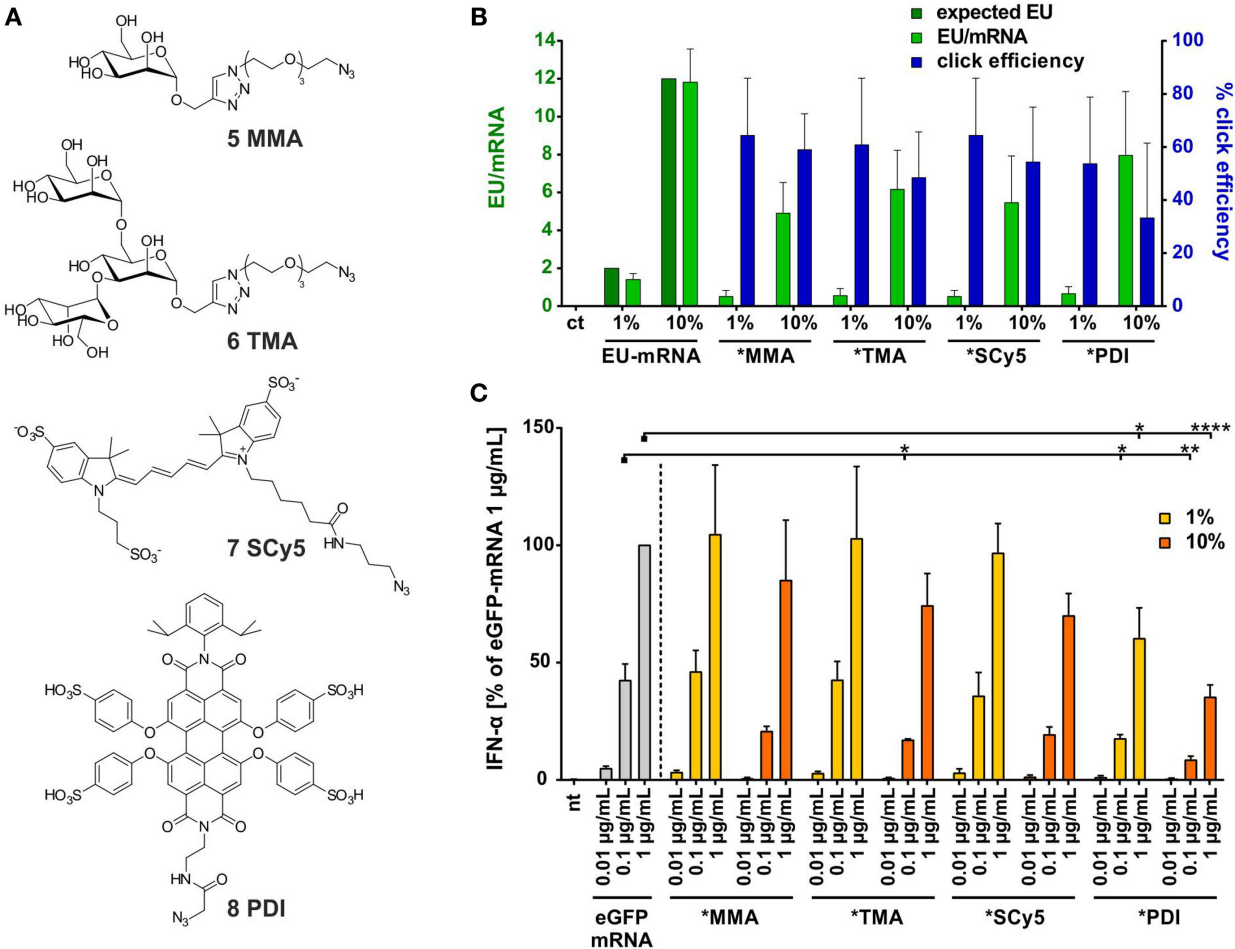
**(A)** Selected azide-bearing molecules of different molecular weight: sugar moieties mono- and trimannose (5, 6), fluorescent dyes Sulfo-Cy5 (7), and perylene-derivative PDI (8). **(B)** HPLC quantification of EU content (light green) in *in vitro* transcription- and clicked-eGFP-mRNA including click efficiency (blue) (*n* = 2–5; mean + SD) (ct = unmodified control eGFP-mRNA). **(C)** Titration of PBMCs with eGFP-mRNA, 1 and 10% alkyne-eGFP-mRNA clicked with molecules 5–8 (nt, non-treated). IFN-α production was measured by ELISA as technical duplicate of biological triplicates (three donors). Due to donor variation in the absolute amount of IFN-α secreted, data from each individual were normalized to 1.0 μg/mL eGFP-mRNA (=100%) of the respective donor (*n* = 3; mean + SD). [Asterisks above bars indicate the respective *P* values evaluated by ANOVA and Sidak’s multiple comparisons test; no declaration = not significant (ns).].

Since the mRNA bioconjugates tested so far all contained substructures known to interact with TLR7, we decided to expand the scope of these investigations to include structures that are *bona fide* non-PAMPs. Figure 5A shows four azides employed in this perspective, which did indeed not cause any IFN-α secretion in stimulation tests (not shown). Two are highly hydrophilic sugar moieties of divergent size, and two are fluorescent dyes of planar structure, whose lipophilicity is partially mitigated by sulfonyl groups. Synthesis of mRNA conjugates was performed as above. Click yields ranged from 50 to 60%, corresponding to 1 or 6 clicked moieties per mRNA molecule, for 1 and 10% EU content, respectively (Figure 5B). As detailed in Figure 5C, the corresponding mRNA conjugates showed a dampened immune response, although to varying degrees. As before, any observable effects increase with the number of attached moieties. The most pronounced effect was seen for the PDI dye, which also has the largest molecular weight. This conjugate is also the only one with a clear effect at 1% EU.

### Transfer to siRNA

**Figure 6:**
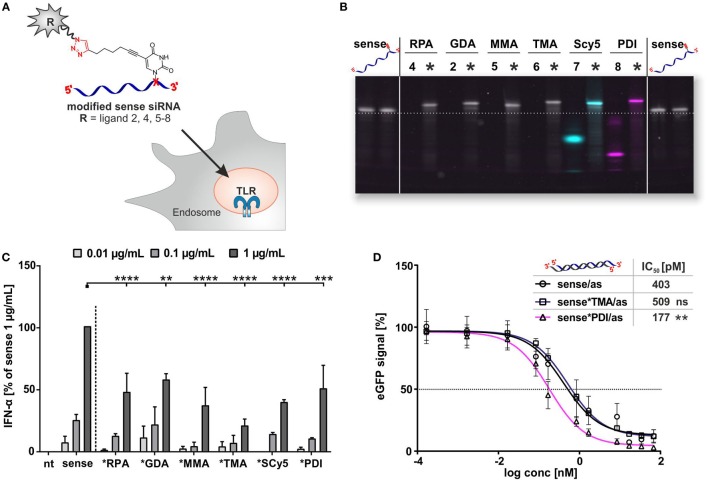
**Application on commercial mono-alkyne-functionalized sense eGFP-siRNA**. **(A)** Sense siRNA constructs for PBMC stimulation. **(B)** Denaturing polyacrylamide gel electrophoresis for comparison of the click efficiency of the single-stranded, alkyne-modified oligonucleotide MH662 (sense), free azides (**4, 2, 5–8**), and purified clicked (*) oligonucleotides, showing band shift after click reaction and additional fluorescent signals for SCy5 (blue) and PDI (purple). Before staining, excitation for dye-carrying constructs was done at 532 nm (PDI) and 633 nm (SCy5). Stains-all (gray/633 nm) was used as loading control to visualize non-fluorescent bands. Emission signals were recorded at 610BP30 nm and 670 nm (superimposition shown). **(C)** Titration of PBMCs with sense siRNA, clicked with molecules 2, 4, and 5–8 (nt, non-treated). IFN-α production was measured by ELISA as technical duplicate of biological triplicates (three donors). To account for donor variation in the absolute amount of IFN-α secreted, data from each individual were normalized to 1.0 μg/mL unmodified siRNA MH662 (=100%) of the respective donor (*n* = 3; mean + SD). (Asterisks above bars indicate *P* values evaluated by ANOVA and Sidak’s multiple comparisons test; no declaration = ns.) **(D)** eGFP-knockdown experiments with unlabeled control- (sense), TMA (6)- and PDI (8)-siRNA double strands [with antisense-strand MH533 (as)] in HeLa MAZ (stably expressing eGFP). IC_50_ (*n* = 3; mean + SD). (Respective *P* values for IC_50_ were evaluated by ANOVA and Dunnett’s multiple comparisons test.)

The above findings suggest that conjugation of small molecules to mRNA reduces the potency of RNA to trigger TLR7-mediated IFN-α secretion and that the degree of reduction depends on the size and the number of small molecules attached to the RNA. This implies a certain dependence on the modification density, i.e., the number of conjugation sites per length unit of the RNA. Consequently, the effect would be expected to be more pronounced even for single attachment sites on smaller RNAs such as siRNAs. We therefore synthesized siRNA conjugates by CuAAC using the same azides as before (Figures 2 and 5). We used an siRNA sequence that previously had been shown to stimulate TLR7 (42). In contrast to mRNA, siRNA conjugates had the additional advantage that they could be separated from unreacted material, hence the immunostimulation data can be attributed to molecules carrying exactly one conjugation site per 22 nucleotides ssRNA, illustrated in Figure 6A. The purified sense strands (Figure 6B) were tested for IFN-α secretion as described before. As shown in Figure 6C, the alkyne-bearing control sense strand (MH662) gives the most prominent amplitude in IFN-α secretion at a concentration of 1 μg/mL. In contrast, all siRNA conjugates show at least a decrease to 55% in TLR7 activation, with the strongest outcome being a sixfold reduction to 20% for the TMA conjugate.

### Influence of RNA Modification on Biologic Activity

The biological activities of both types of RNA after CuAAC conjugation were investigated bearing in mind that both are being actively investigated as therapeutic agents. Translation efficacies of click-conjugated mRNA derivatives were compared to their untreated controls by measuring the fluorescence of the encoded reporter protein eGFP. Therefore, immature DCs were electroporated with differentially treated mRNA samples. Fluorescence intensity was measured 24 h later by flow cytometry. The introduction of an alkyne moiety *via* IVT did not have any negative impact on protein expression at neither 1% (Figure S19 in Supplementary Material) nor 10 EU% (not shown). However, CuAAC-mediated conjugation of any azide compound featured in Figures 2 and 5 ablated translational activity completely. Testing of material from mock reactions, i.e., click reactions without azide compound, confirmed that this effect is due to the conjugation and not a consequence of the reaction conditions of the CuAAC (Figure S19 in Supplementary Material). We conclude that even a single lateral conjugation anywhere onto an mRNA is incompatible with the translation apparatus.

In order to assess how the respective bioconjugations would influence the RNAi efficiency of an siRNA, IC_50_ values were determined in HeLa MAZ cells (59) *via* the knockdown of a destabilized eGFP with selected constructs (Figure 6D). After hybridization of clicked sense-strand derivatives to the appropriate antisense strand, cells were incubated with a concentration series of siRNA double strands and eGFP fluorescence emission measured by FACS 24 h later. In keeping with our previously reported identification of a permissive attachment site on the 3′-end of the sense strand (70), conjugation of various azides did not significantly increase the IC_50_ values (Figure 6D). Indeed, the *PDI derivative (pink in Figure 6) showed an IC_50_ value improved by ~3-fold.

### The Effect of Bioconjugates on TLR7-Mediated Immunostimulation Is Distinct from Inhibition by Ribose Methylation

**Figure 7:**
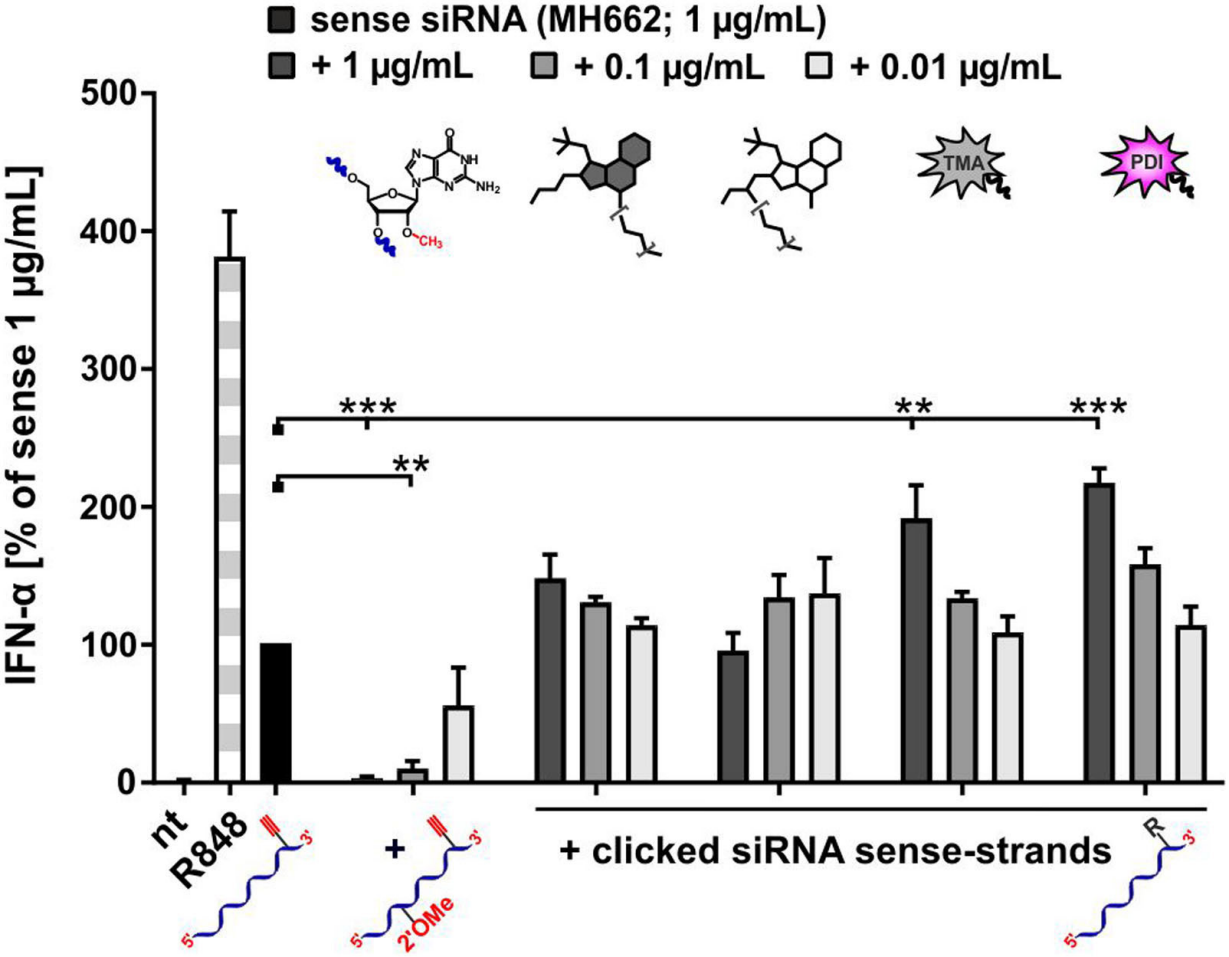
**A new distinct type of prevention of toll-like receptor 7-mediated immunostimulation: steric shielding acts different from ribose methylation**. PBMCs were incubated with 1.0 μg/mL sense siRNA (MH662) and simultaneously with a titration series of MH662 bearing either a Gm motive (2′-OMe) at position 8, or MH662 clicked to azides of resiquimod-polyethylene-glycol-azide (4), gardiquimod-diethylene-glycol-azide (2), TMA (6), or PDI (8) (nt, non-treated). IFN-α production was measured by ELISA as technical duplicate of biological triplicates (three donors). To account for donor variation in the absolute amount of IFN-α secreted, data from each individual were normalized to 1.0 μg/mL unmodified siRNA MH662 (=100%) of the respective donor (*n* = 3; mean + SD). (Asterisks above bars indicate the respective *P* values evaluated by ANOVA and Dunnett’s multiple comparisons test; no declaration = ns.)

Given that the bioconjugates of smTLRa ligands, as well as all other conjugates showed a decreased stimulation of TLR7, the question arose, if this decrease was comparable to that known from RNA carrying a Gm residue. This residue, a G nucleotide with a 2′-*O*-methylation, when placed in the right sequence context, was previously shown to act as a TLR7 antagonist when applied together with otherwise stimulatory RNA (33, 35). A corresponding assay was carried out with four of the above siRNA conjugates, namely, of RPA, GDA, TMA, and PDI. A constant concentration of stimulatory siRNA was co-incubated with increasing amounts of the conjugates, and for comparison an siRNA carrying a Gm modification was investigated under the same conditions. Figure 7 shows a striking and clear inhibition of the Gm-RNA in comparison to the stimulatory RNA alone. In contrast, all mixtures of stimulatory RNA and conjugates showed a moderate and concentration dependent increase of IFN-α emission relative to the standard, presumably as a result of an overall increased amount of applied RNA. Hence, the effect of bioconjugation on stimulation of TLR7 is neither inhibitory, nor as pronounced as that of Gm.

## Discussion

The work described here was based on the working hypothesis that TLR7 activation might be synergistically increased by the combination of two known but distinct TLRa, namely, ssRNA and small molecules of the -quimod series. Previous work by the Weber group provided experimental evidence that these two classes of TLRa engage TLR7 in different recognition modes, since they lead to different signaling cascades (27), which could be confirmed by resent investigations on TLR7-crystal structures by Zhang et al. (28). Our attempts to unite both patterns in a single bidentate molecular entity clearly failed, since the covalent conjugates of resiquimod to RNA decreased the TLR7-mediated interferon response, rather than increasing it. One interesting result is contained within the control reactions performed in this context, though: combined administration of mRNA and (unconjugated) resiquimod can still increase the interferon response that was already saturated with respect to resiquimod—compare light blue and dark blue bars in the R848 panel in Figure 4. This indicates additional capacity for activation not accessible by R848 alone. Synergistic but also anti-synergistic effects of stimulation by nucleic acid in combination with imidazoquinolines have been described for DNA in the context of human and murine TLR7/8 systems (71–73). For example, poly(T) ODNs inhibited TLR7 activation but enhanced TLR8 signaling by imidazoquinoline derivatives optimized to trigger either TLR7 or TLR8. Those effects seemed to be independent from DNA receptor TLR9. Here, experiments with GDA, a non-stimulatory derivate, show that the opposite also occurs: coupling GDA to mRNA (Figure 4) inhibited activation by otherwise stimulatory RNA nucleic acid.

This modification scheme by CuAAC chemistry on mRNA was explored, to our best knowledge, for the first time concerning immunostimulation and protein expression. The complete ablation of mRNA translation on the 5-position of uridines, even by a single modification, is somewhat surprising. It suggests that, besides the coding region, the entire length of the RNA (with the possible exception of the poly-A tail) is subject to some kind of steric surveillance, which is unlikely to be effected by ribosomes alone, since the 3′-UTR is also concerned. More promising is the continued function of siRNA conjugates, whose potentially undesired immunostimulation can be partially shielded by bulky conjugates. A serendipitous discovery is the actual improvement of RNAi efficiency upon addition of the large perylene dye. While one might speculate this to be related to issues of membrane penetration from the endosomal compartment into the cytosol, detailed follow-up studies are required to determine the extent of this effect.

The recurrent observation of a moderately decreased response to conjugates containing small molecules attached to the RNA chain laterally (mRNA) or terminally (siRNA) are in contrast to our previous observations of a truly active antagonist mode displayed by naturally occurring ribose methylations in defined nucleotide contexts, which, despite being much smaller, block activation even in the presence of otherwise potently stimulatory RNA (33, 35). Because we have ruled out a similar effect for the RNA bioconjugates synthesized here (see comparative data in Figure 7), we conclude that it is likely that the bioconjugated small molecule residues provide some amount of steric shielding to the RNA, reducing TLR activation simply by blocking access to recognition notices in the RNA proper. In contrast, the antagonistic action of ribose-methylated RNAs is more in keeping with a mechanism in which the methylated RNA is bound by TRL7 but inhibits a conformational rearrangement conductive to signaling. Given that our work failed to identify a new structural principle for the activation of TLR7, we feel that it would mean over-interpretation to excessively discuss solely negative data in the context of the published X-ray structures of TLR/and TLR8 (28, 29).

In summary, in failing to show cooperative TLR7 stimulation by R848-RNA conjugates, we have described a general steric shielding effect to reduce TLR7 stimulation by RNA. Our concluding experiment (Figure 7) has shown that the steric shielding effect discovered here is of fundamentally different nature than the inhibition known from ribose methylation (33, 35). Although, the latter is already an elegant method to prevent RNA molecules from immunostimulation, our findings are by no means negligible in RNA-bioconjugate chemistry as any label may potentially influence TLR7 stimulation. Of note, siRNA conjugates to trimeric sugar moieties similar to the trimannose conjugate (*TMA in Figures 5 and 6) are in preclinical trials (74), and their immunogenic potential is likely to be affected in a similar way.

## Author Contributions

IH—contributions to design of the work, performed research, acquisition and analysis of data, illustration, initial drafting of the manuscript, critical revision, and final approval; IF—performed research, acquisition and analysis of data, critical revision, and final approval; JS, SS-L, KT, KS, JL, SK, MK, and SR—performed research and final approval; TE—contributions to critical discussion and analysis of data and final approval; KP—contributions to design of the work, supervision, and final approval; TO and HJ—contributions to design of the work, critical revision, supervision, and final approval; AD—contributions to design of the work, illustration, critical revision, supervision, and final approval; MH—design of the work, illustration, writing of the manuscript, critical revision, supervision, and final approval.

## Conflict of Interest Statement

The authors declare that the research was conducted in the absence of any commercial or financial relationships that could be construed as a potential conflict of interest.
