# Supplementary material for: Bioconjugation of Small Molecules to RNA Impedes Its Recognition by Toll-Like Receptor 7
Source: Front Immunol. 2017 Mar 24;8:312. doi: 10.3389/fimmu.2017.00312 (PMC5364167; doi:10.3389/fimmu.2017.00312)
Supplement: Supplementary file 1 [file Data_Sheet_3.docx]

Supplementary Material

Bioconjugation of Small Molecules to RNA Impedes Its Recognition by Toll-Like Receptor 7

Isabell Hellmuth^1^, Isabel Freund^2^, Janine Schlöder^3^, Salifu Seidu-Larry^1^, Kathrin Thüring^1^, Kaouthar Slama^1^, Jens Langhanki^4^, Stefka Kaloyanova^5^, Tatjana Eigenbrod^2^, Matthias Krumb^4^, Sandra Röhm^5^, Kalina Peneva^5^, Till Opatz^4^, Helmut Jonuleit^3^, Alexander H. Dalpke^2^ and Mark Helm^1^*

^1^Institute of Pharmacy and Biochemistry, Johannes Gutenberg-University Mainz, 55128 Mainz, Germany.

^2^Department of Infectious Diseases, Medical Microbiology and Hygiene, University of Heidelberg, 69120 Heidelberg, Germany.

^3^Department of Dermatology, University Medical Center of the Johannes Gutenberg-University Mainz, 55131 Mainz, Germany.

^4^Institute of Organic Chemistry, Johannes Gutenberg-University Mainz, 55128 Mainz, Germany.

^5^Max Planck Institute for Polymer Research, 55128 Mainz, Germany.

*** Correspondence**: Tel: +49 6131 39 25731; Fax: +49 6131 39 20373; Email: [mhelm@uni-mainz.de](mailto:mhelm@uni-mainz.de)

Table of contents

[1 General Experimental 4](#_Toc477101497)

[2 Synthesis of azide-modified TLR ligands 6](#_Toc477101498)

[2.1 Spectra obtained for azide-modified TLR ligands 8](#_Toc477101499)

[3 Synthesis of azide-modified saccharides 12](#_Toc477101500)

[3.1 Spectra obtained for azide-modified saccharides 21](#_Toc477101501)

[4 Synthesis of azide-modified perylene dye 23](#_Toc477101502)

[4.1 Spectra obtained for azide-modified perylene dye 24](#_Toc477101503)

[5 In addition to figure 2C: 25](#_Toc477101504)

[6 Cytotoxicity assay of commercial small molecules and azide-derivatives in PBMCs 26](#_Toc477101505)

[7 RNA sequence information 27](#_Toc477101506)

[8 Tritium incorporation assay of 5’-mRNA capping reaction 28](#_Toc477101507)

[9 mRNA integrity after click-reaction 29](#_Toc477101508)

[10 Comparison of the effect of smTLRa, RNA, and covalent conjugates of both in immunostimulation. 30](#_Toc477101509)

[11 eGFP expression in human immature DC 31](#_Toc477101510)

[12 References 32](#_Toc477101511)

# General Experimental

All reagents were reagent grade and used without further purification, unless otherwise noted. Gardiquimod was purchased from Enzo Life Sciences (Lörrach, Germany) and resiquimod from InvivoGen (San Diego, California USA). Dimethylformamide (DMF, Extra dry, AcroSeal ®) and pyridine was purchased from Acros Organics (Belgium) and used without further purification. Acetonitrile and dichloromethane (DCM) were distilled from calcium hydride. The eluents for column chromatography were distilled prior to use. Deuterated solvents were purchased from Deutero GmbH (Kastellaun, Germany). All reactions involving air or moisture sensitive reagents or intermediates were performed under an inert atmosphere of argon in glassware that was oven dried using standard Schlenk techniques. Reaction temperatures referred to the temperature of the particular cooling/heating bath.

**Thin layer chromatography (TLC)**: Pre-coated silica gel plates, Polygram^®^ Sil G/UV_254_ (40 x 80 mm) from Macherey-Nagel (Düren, Germany) or RP silica gel RP-18 F_254s_ plates (Merck, Darmstadt, Germany) were used for monitoring chemical reactions. Compound spots were visualized with UV-light at λ = 254 nm and/or by immersion in Seebach’s reagent, consisting of phosphor-molybdic acid (2.50 g), ceric sulfate Ce(SO_4_)_2_ (1.00 g) and concentrated sulfuric acid H_2_SO_4_ (6 mL in 94 mL of water) followed by heating. Alternatively, the TLC plates were developed in an iodine-chamber or immersed in a solution of *m*-methoxyphenol (0.1 mL) in ethanol (95 mL) and sulfuric acid (2 mL) followed by heating.

**Column chromatography**: Silica gel 60 (230-400 mesh) was purchased from Sigma-Aldrich (Taufkirchen, Germany; product manufactured by Fluka, Buchs, Switzerland) and Merck (Darmstadt, Germany), respectively. Further chromatography was performed using flash chromatography of the indicated solvent system on 35‑70 µm silica gel (Acros Organics) unless otherwise noted. Alternatively, the purifications were performed on an Isolera™ Flash Purification System (Biotage®) with an integrated diode array detector.

Preparative reverse phase separation was carried out on a *Smartline* HPLC system (Knauer, Berlin, Germany) with mixtures of acetonitrile or methanol and water as eluents. The eluents were degassed prior to use by means of ultrasonication for 30 min. Two *Smartline K-1800* pumps (pump head size: 100 mL each, high pressure gradient mode; Knauer) and an *S-2600 diode array detector* (Knauer) were used.

**Mass spectrometry**: Mass spectra were obtained from various instruments depending on the required technology: ESI spectra: Micromass LCT spectrometer; High-resolution masses (ESI) were recorded on a *Q-ToF-Ultima 3* instrument (Waters) with LockSpray^®^ interface and a suitable external calibrant. Further ESI measurements were received from a Finnigan MAT TSQ 700 and FD results from a Finnigan MAT95. MALDI-TOF mass spectra were recorded on a Bruker BIFLEX III.

**UV/VIS** spectra were recorded on a Perkin-Elmer Lambda 9; **fluorescence** spectra on SPEX Fluorolog 3 spectrometer.

**NMR-spectroscopy**: NMR spectra were recorded on an *Avance III HD 300* (300 MHz ^1^H-NMR, 75 MHz ^13^C-NMR; Bruker), an *Avance II 400* (400 MHz ^1^H-NMR, 101 MHz ^13^C-NMR; Bruker), an *Avance III 600* (600 MHz ^1^H-NMR, 151 MHz ^13^C-NMR; Bruker) or *Avance 700* (700 MHz ^1^H-NMR, 175 MHz ^13^C-NMR; Bruker) using 5 mm probe heads at a temperature of 23 °C. The ^13^C-NMR spectra are ^1^H broadband decoupled. The ^1^H and ^13^C chemical shifts (δ) were referenced to the residual solvent signal as internal standard (CDCl_3_: δ = 7.26 ppm and 77.16 ppm, DMSO-*d*_6_: δ = 2.50 ppm and 39.50 ppm, CD_3_OD: δ = 3.31 ppm and 49.00 ppm for ^1^H and ^13^C NMR, respectively) (1). Coupling constants (*J*) are reported in Hz (splitting abbreviations: s, singlet; d, doublet; t, triplet; q, quartet; m, multiplet; br, broad; and combinations thereof).


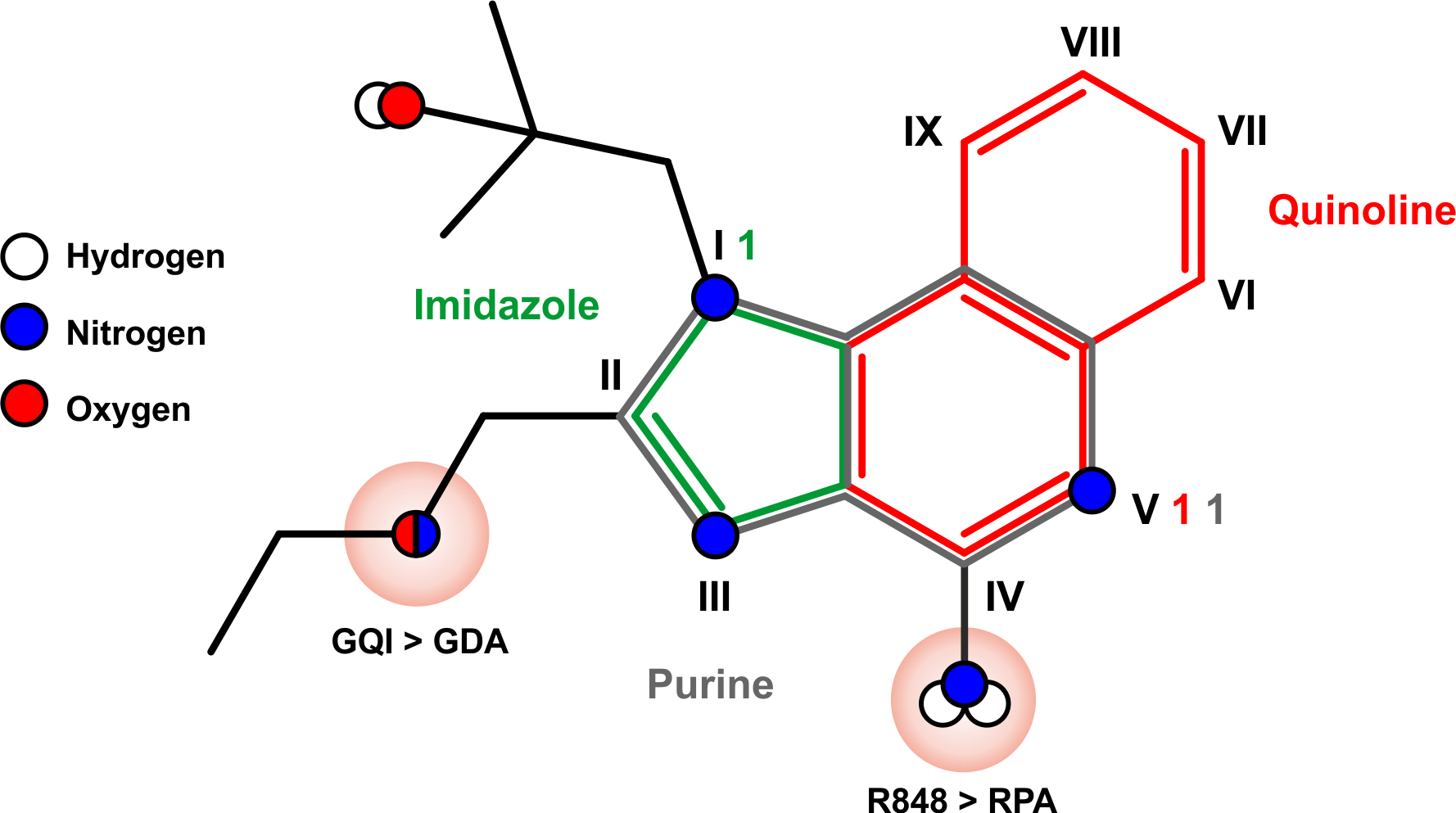


**Supplementary Figure 1.** *Numbering of imidazoquinoline based small molecule scaffolds for nomenclature.* Atoms of the overall system are counted in roman numerals. Syntheses starting points are highlighted within orange circles.

# Synthesis of azide-modified TLR ligands

**Synthesis of 2-(2-(2-azidoethoxy)ethoxy)ethyl methanesulfonate II**

2-[2-(2-Azidoethoxy)ethoxy]ethanol **I** (200 mg, 1.14 mmol, 1.0 eq.) was dissolved in molecular sieve-dried dichloromethane (2.0 mL). The mixture was stirred and cooled to 0 °C after which methanesulfonyl chloride (157 mg, 1.37 mmol, 1.5 eq.) was added as a solution in dry DCM. The catalyst trimethylamine (231 mg, 2.28 mmol, 2.0 eq.) was then added in drops after which the reaction mixture was allowed to warm to room temperature. Stirring for 1 h led to complete conversion, monitored on TLC-plate (DCM/MeOH, 9:1) *via* iodine-staining. After removing the solvent *in vacuo*, the crude mixture was purified *via* column chromatography by using DCM and 10 % MeOH as eluent, giving the title compound **II** (230 mg, 0.91 mmol, 80 %) as a yellowish oil. *R_f_* = 0.28 (silica gel, DCM/MeOH, 9:1).

^1^H-NMR (300 MHz, DMSO-*d6*) δ [ppm] = 4.30 (t, ^3^*J* = 8.8 Hz, ^3^*J* = 4.4 Hz, 2H, CH_2_), 3.68 (t, ^3^*J* = 8.8 Hz, ^3^*J* = 4.4 Hz, 2H, CH_2_), 3.62-3.58 (m, 6H, 3 x CH_2_), 3.39 (t, ^3^*J* = 9.8 Hz, ^3^*J*= 4.9 Hz, 2H, CH_2_), 3.17 (s, 3H, CH_3_). ^13^C-NMR (75 MHz, DMSO-*d6*) δ [ppm] = 69.75 (*C*H_2_), 69.64 (*C*H_2_), 69.60 (*C*H_2_), 69.24 (*C*H_2_), 68.32 (*C*H_2_), 49.97 (*C*-N_3_), 36.78 (*C*H_3_).

**Synthesis of 1-(4-amino-2-(((2-(2-(2-azidoethoxy)ethoxy)ethyl)(ethyl)amino)methyl)-1H-imidazo[4,5-c]quinolin-1-yl)-2-methylpropan-2-ol (Gardiquimod-DEG-N_3_ / GDA) 2**

Gardiquimod **1** (37.6 mg, 0.12 mmol, 1 eq.) and compound **II** (50.0 mg, 0.19 mmol, 1.6 eq.) were dissolved in dry *N,N’*-dimethylformamide (10.0 mL), giving a clear solution. The reaction mixture was stirred over night at room temperature. Monitoring by TLC (DCM/MeOH, 7:3) showed an estimated conversion of 60 %. After removing the solvent *in vacuo*, the crude mixture was purified *via* column chromatography by using a gradient system of DCM and 10-30 % MeOH as eluent, giving the title compound **2** (28 mg, 0.06 mmol, 50 %) as a white powder. *R_f_* = 0.33 (silica gel, DCM/MeOH, 7:3).

^1^H-NMR (300 MHz, MeOD) δ [ppm] = 8.24 (d, ^3^*J* = 8.2 Hz, 1H, H-Ar), 7.66 (d, ^3^*J* = 8.3 Hz, 1H, H-Ar), 7.47 (t, ^3^*J* = 15.3 Hz, ^3^*J* = 7.6 Hz, 1H, H-Ar), 7.32 (t, ^3^*J* = 15.2 Hz, ^3^*J* = 7.6 Hz, 1H, H-Ar), 4.90 (s, 2H, C*H_2_*-N), 3.57 (t, ^3^*J* = 10.6 Hz, ^3^*J* = 5.3 Hz, 2H, CH_2_), 3.52-3.49 (m, 6H, 3 x CH_2_), 3.27-3.21 (m, 6H, 2 x CH_2_, -NH_2_), 2.76 (t, ^3^*J* = 10.9 Hz, ^3^*J* = 5.4 Hz, 2H, CH_2_), 2.71-2.64 (m, 3H, C*H*_2_-CH_3_, -OH), 1.24 (br s, 6H, 2 x CH_3_), 1.08 (t, ^3^*J* = 14.1 Hz, ^3^*J* = 7.1 Hz, 3H, CH_2_-C*H*_3_). ^13^C-NMR (75 MHz, MeOD) δ [ppm] = 154.1, 152.6, 144.2, 136.9, 128.8, 126.6, 125.8, 123.4, 122.5, 116.4, 72.2, 71.4, 71.3, 71.0, 70.2, 56.7, 53.6, 52.9, 52.8, 51.7, 46.7, 39.4, 11.4. HR-MS (ESI): m/z calculated for [C_23_H_34_N_8_O_3_ + H]^+^: 471.2832; found: 471.2832.

**Synthesis of 1-azido-N-(2-(ethoxymethyl)-1-(2-hydroxy-2-methylpropyl)-1H-imidazo[4,5-c]quinolin-4-yl)-3,6,9,12-tetraoxapentadecan-15-amide (Resiquimod-PEG_4_-N_3_ / RPA) 4**

Resiquimod **3** (22 mg, 70 µmol, 1 eq.) was dissolved in 4.0 mL dry dichloromethane. After the addition of 2,5-dioxopyrrolidin-1-yl-1-azido-3,6,9,12-tetraoxapentadecan-15-oate (NHS-PEG_4_-N_3_) (32 mg, 84 µmol, 1.2 eq.), the reaction mixture was stirred under argon until no further conversion could be observed, monitored *via* TLC (CHCl_3_/EtOH, 9:1). The reaction mixture was extracted three times with water (2.0 mL) in order to get rid of excess starting material **3**, organic phases were combined and concentrated *in* *vacuo* to dryness on a rotary evaporator. For further purification, a column chromatography (silica) with a solvent-gradient 100-88 % CHCl_3_ and 0-12 % EtOH was performed. Fractions containing product were combined and concentrated *in vacuo,* yielding the title compound **4** (9.05 mg, 15.4 µmol, 25 %) as highly viscous colorless oil. *R_f_* = 0.33 (silica gel, CHCl_3_/EtOH, 9:0.5).

^1^H-NMR (300 MHz, DMSO-*d6*) δ [ppm] = 8.42 (d, ^3^*J* = 8.2 Hz, 1H, H-Ar), 8.02 (d, ^3^*J* = 8.2 Hz, 1H, H-Ar), 7.70 (t, ^3^*J* = 15.2 Hz, ^3^*J* = 7.5 Hz, 1H, H-Ar), 7.62 (t, ^3^*J* = 15.1 Hz, ^3^*J* = 7.5 Hz, 1H, H-Ar), 4.99 (s, 2H, CH*_2_*-O), 4,81 (s, 2H, imidazo-CH*_2_*), 4.40 (q, ^3^*J* = 7.2 Hz, 1H, NH), 3.97 (t, ^3^*J* = 11.8 Hz, ^3^*J* = 5.9 Hz, 2H, O=C-CH_2_), 3.76 (s, 4H, 2 x CH_2_), 3.27-3.21 (m, 14H, 7 x CH_2_), 3.35 (t, ^3^*J* = 10.1 Hz, ^3^*J* = 5.0 Hz, 2H, CH_2_-N_3_), 3.20 (br s, 1H, -OH), 1.39-1.36 (br s, 6H, 2 x CH_3_), 1.25-1.21 (m, 3H, CH_2_-C*H*_3_). ^13^C-NMR (75 MHz, DMSO-*d6*) δ [ppm] = 172.5, 143.8, 116.2, 77.3, 71.6, 70.8, 70.77, 70.75, 70.7, 70.5, 70.1, 67.0, 66.6, 65.5, 56.7, 50.7, 38.2, 28.1, 25.5, 15.1. HR-MS (ESI): m/z calculated for [C_28_H_41_N_7_O_7_ + Na]^+^: 610.2965; found: 610.2945.

## Spectra obtained for azide-modified TLR ligands
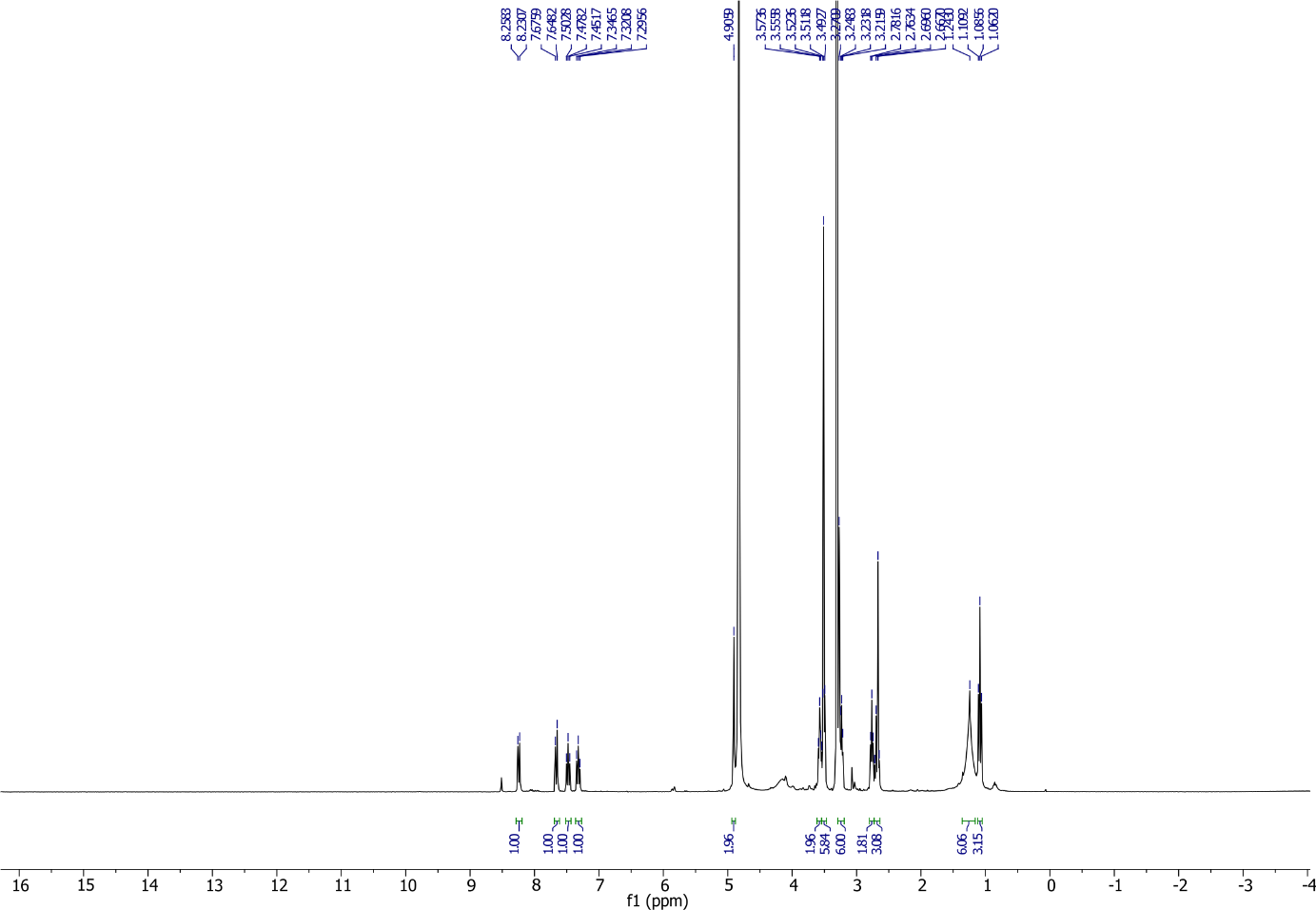


Supplementary Figure 2. ^1^H-NMR (300 MHz, MeOD), GDA 2.


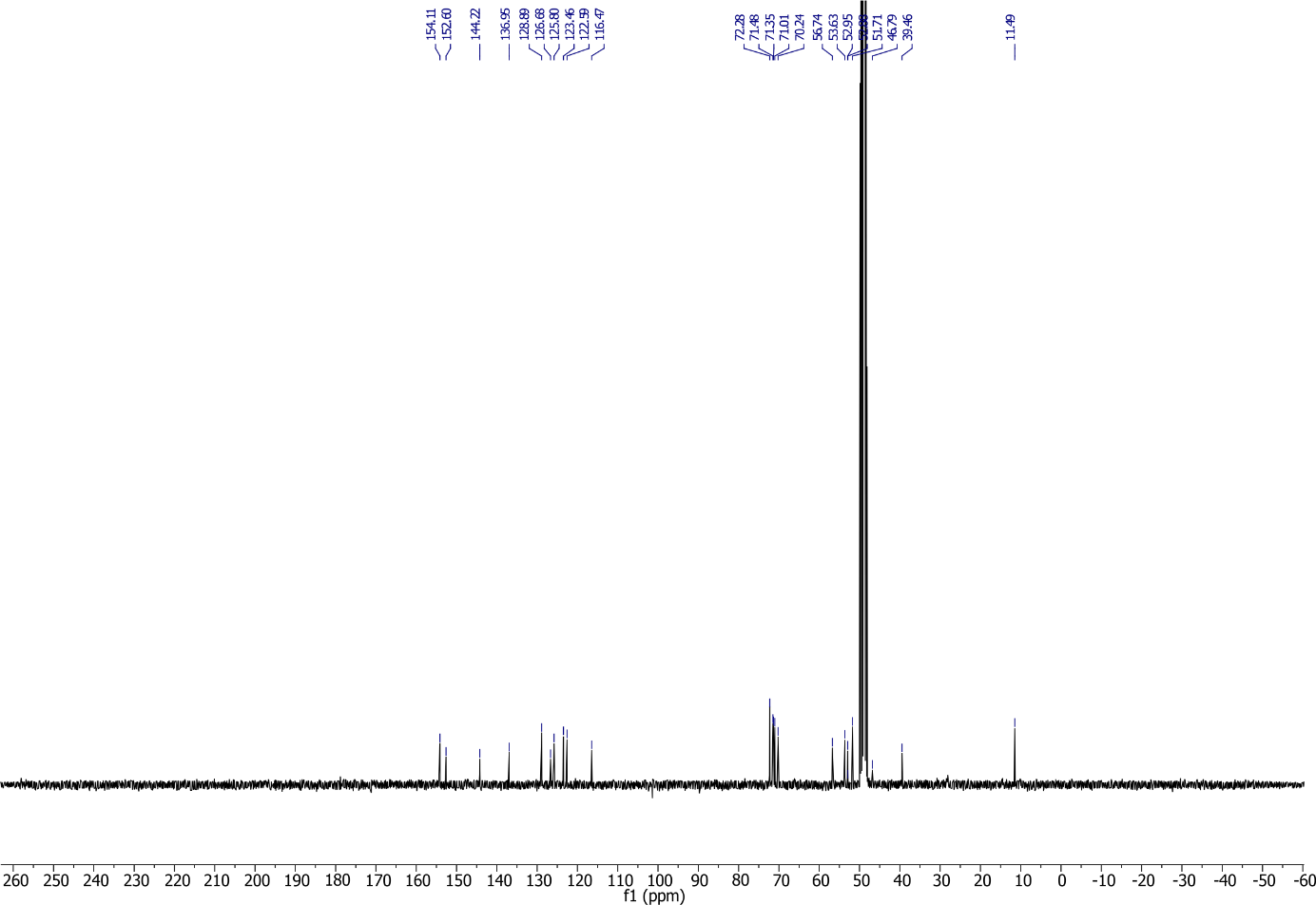
Supplementary Figure 3. ^13^C-NMR (300 MHz, MeOD), GDA 2.


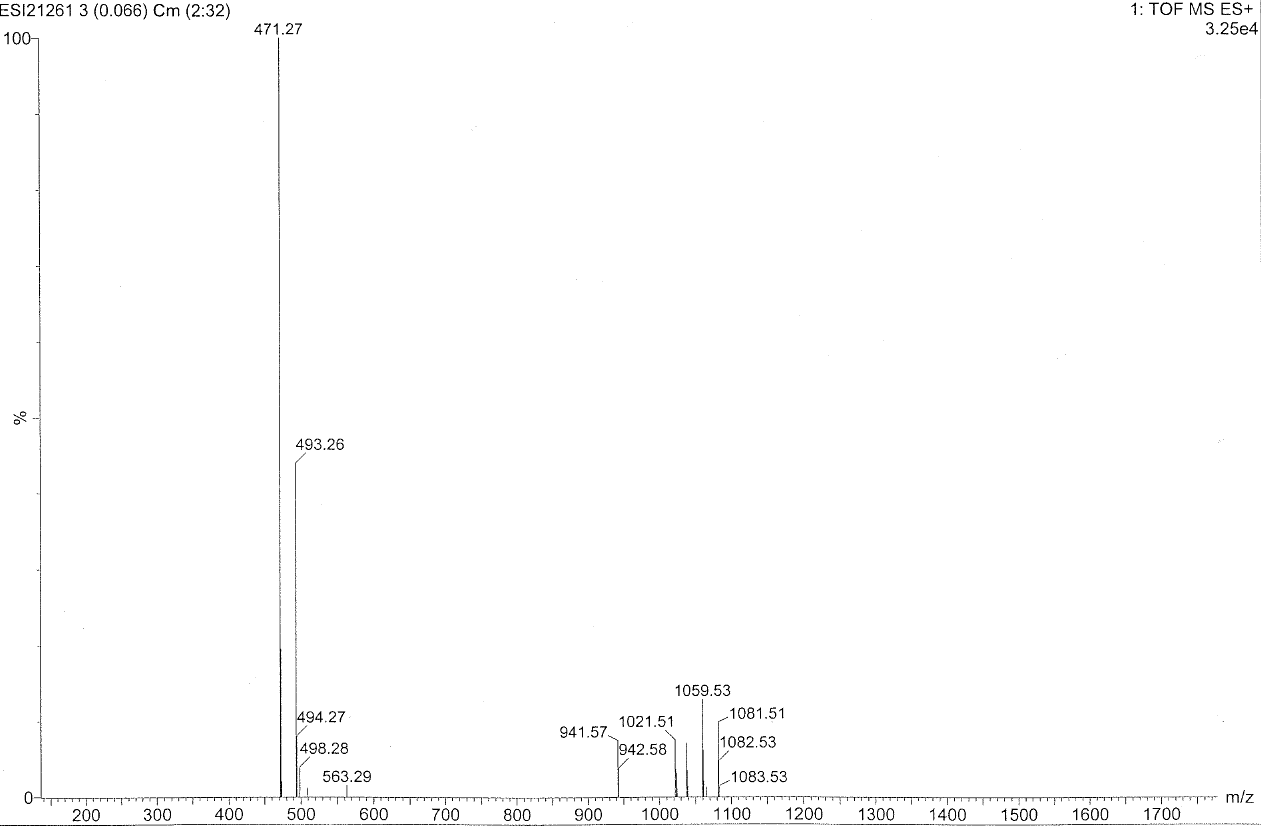


Supplementary Figure 4. Full range MS (ESI) GDA 2.


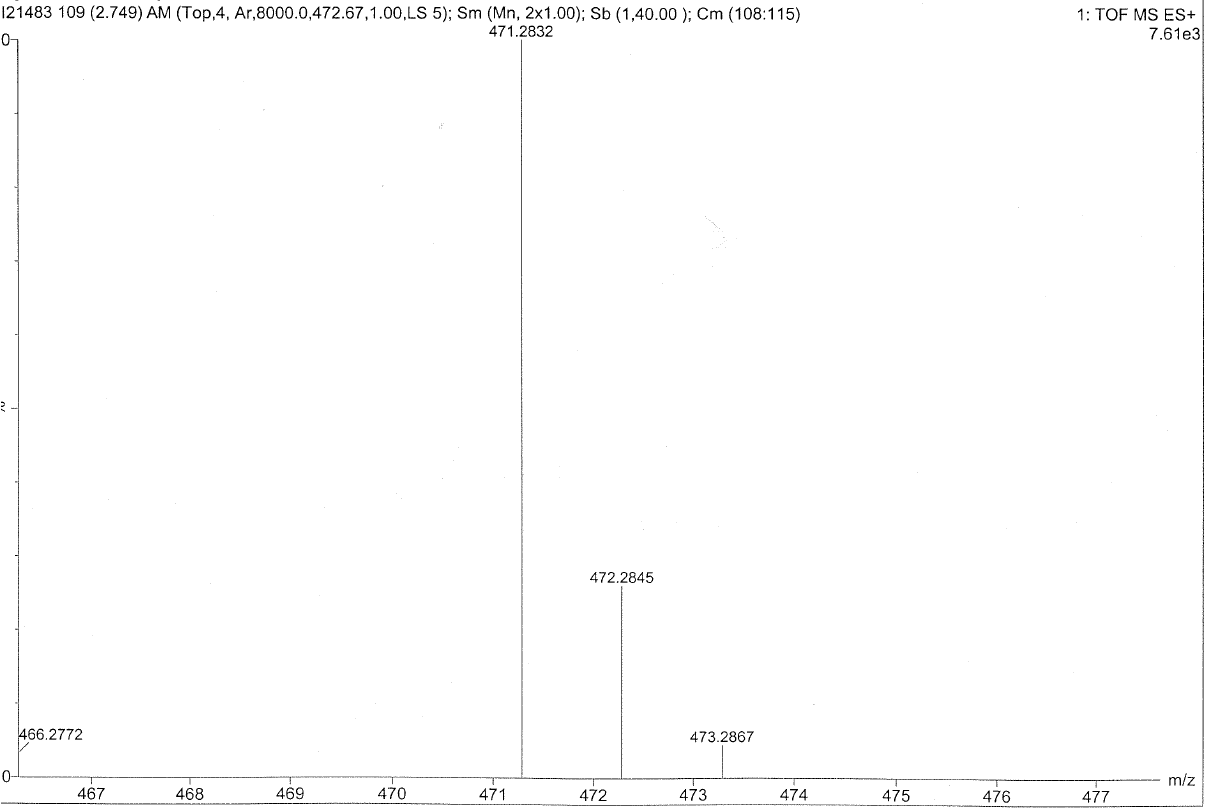
**Supplementary Figure 5.** HR-MS (ESI) GDA **2**.

**
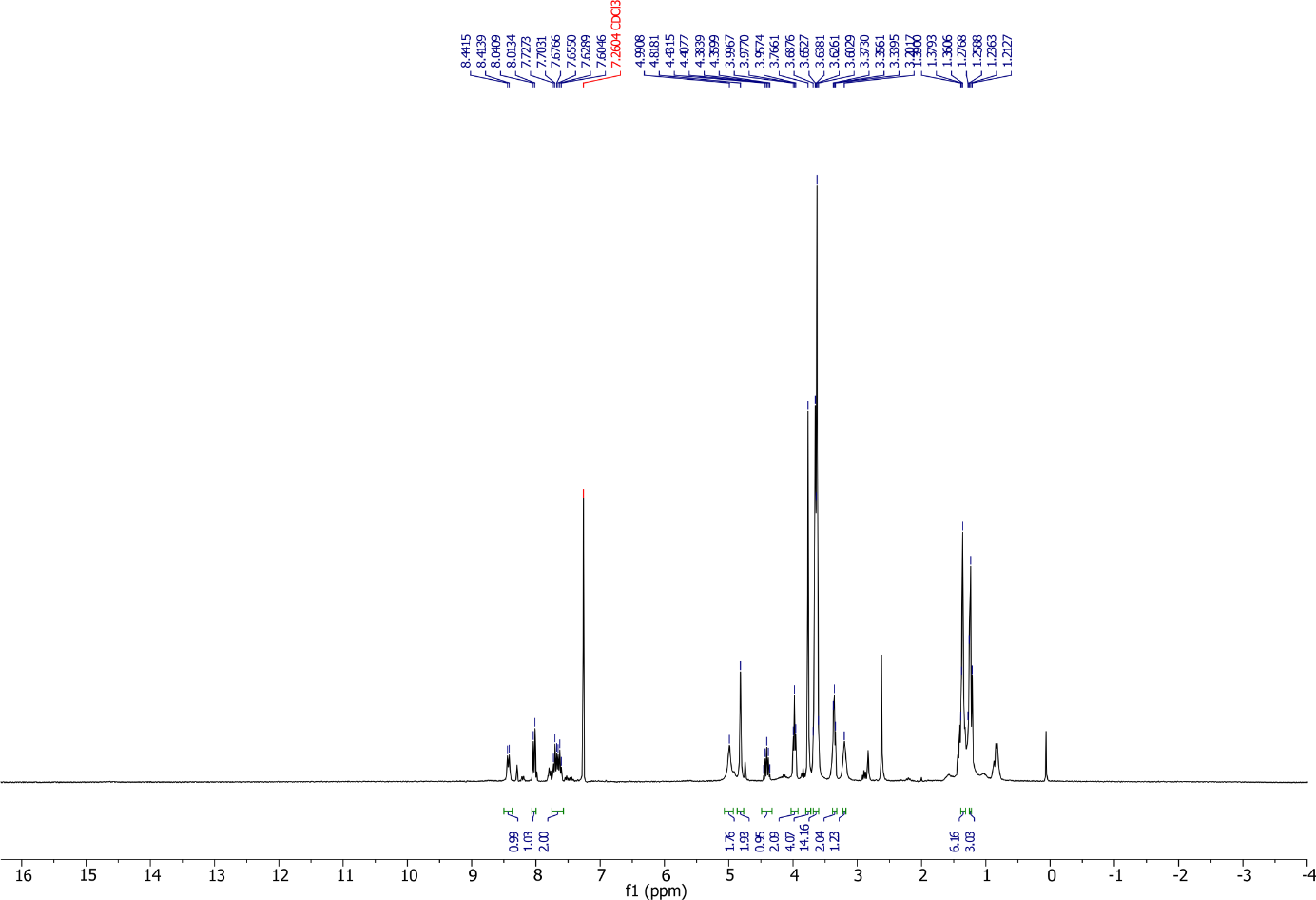
**

Supplementary Figure 6. ^1^H-NMR (300 MHz, CDCl_3_), RPA 4.

**
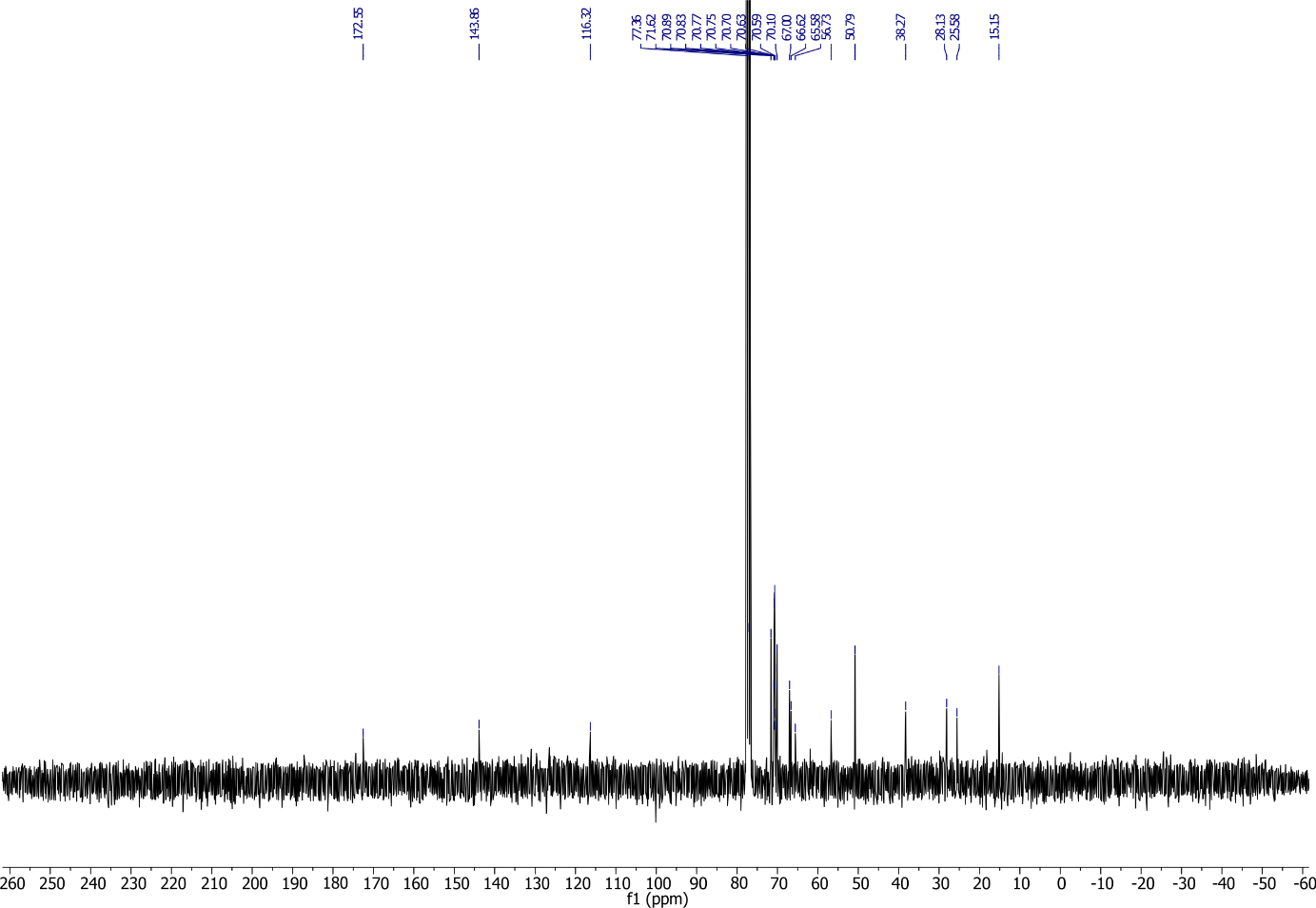
**

Supplementary Figure 7. ^13^C-NMR (300 MHz, CDCl_3_), RPA 4.


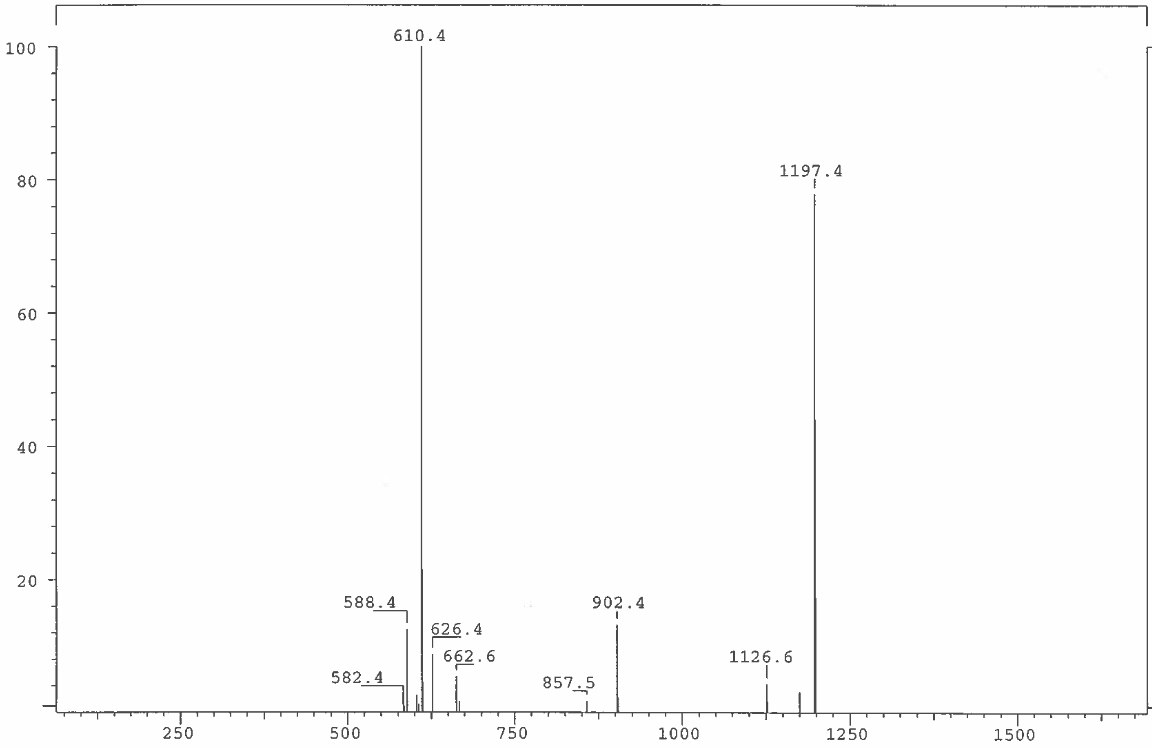


Supplementary Figure 8. Full range MS (FD) RPA 4.


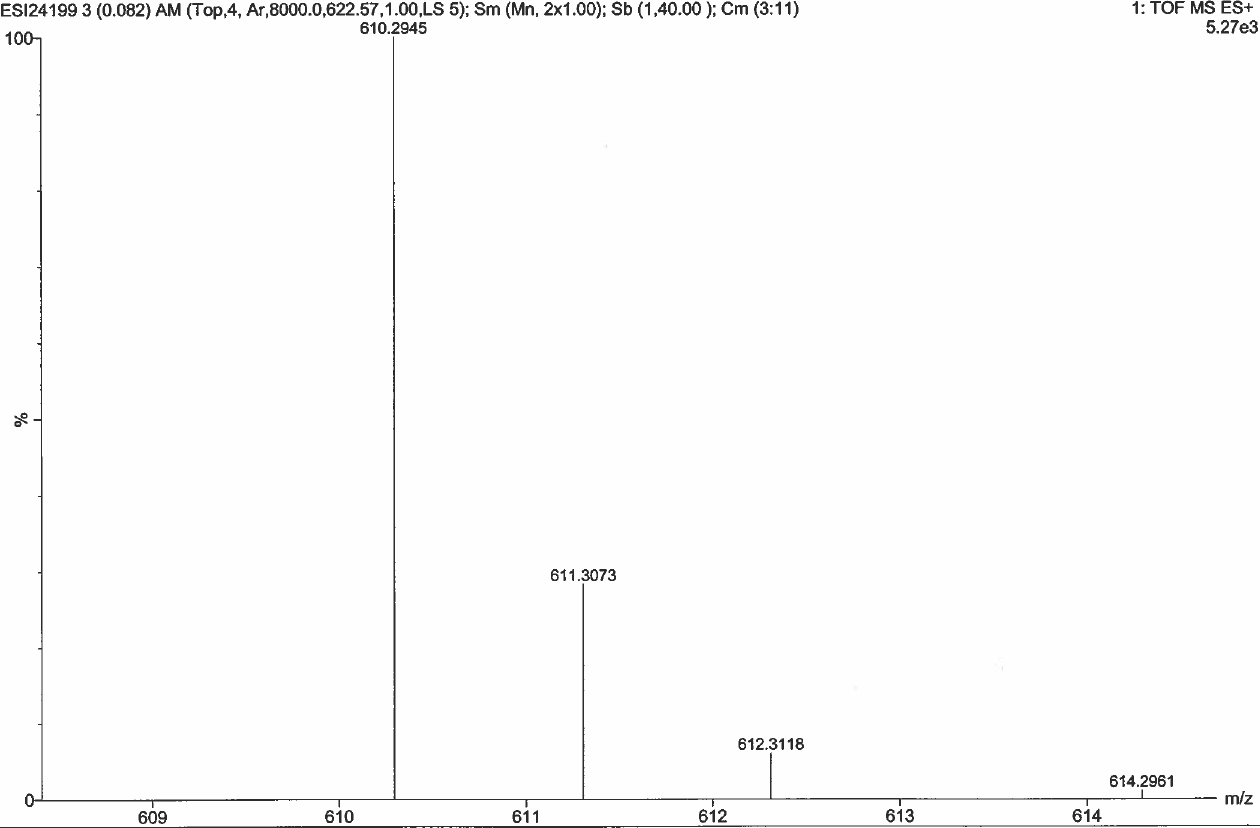


Supplementary Figure 9. HR-MS (ESI) RPA 4.

# Synthesis of azide-modified saccharides

**1,2,3,4,6-Penta-*O*-acetyl-α,β-d-mannopyranose 6**

Iodine (560 mg, 2.2 mmol, 0.04 eq.) and acetic anhydride (50 mL) were mixed under Ar‑atmosphere. d-Mannose **5** (10.0 g, 55.5 mmol, 1 eq.) was added portion by portion at 0 °C. After stirring for 30 min at 0 °C and additionally for 18 hours at room temperature, TLC (cyclohexane/toluene/ethylacetate 3:3:1) showed complete consumption of the starting material. The reaction mixture was diluted with dichloromethane (50 mL) and was washed twice with cold saturated aqueous Na_2_SO_3_ solution (2 × 80 mL), then with a saturated aqueous solution of NaHCO_3_ (4 × 50 mL). The separated organic layer was dried over anhydrous MgSO_4_. The solvent was removed *in vacuo* to afford the desired peracetylated d‑mannose (21.5 g, 55.1 mmol, 99 %, mixture of both anomers α /β 4.75:1) as a yellowish high viscous oil. *R_f_* = 0.30 (silica gel, cyclohexane/toluene/ethyl acetate, 3:3:1);

Signals assignable to α-anomer: ^1^H-NMR (600 MHz, CDCl_3_) δ [ppm] = 6.09 (d, *^3^J* = 1.9 Hz, 1H, H‑1), 5.34–5.36 (m, 2H, H‑3, H‑4), 5.25–5.27 (m, 1H, H-2), 4.28 (dd, *^2^J* = 12.4 Hz, *^3^J* = 4.9 Hz, 1H, H‑6a), 4.10 (dd, *^2^J* = 12.4 Hz, *^3^J* = 2.5 Hz, 1H, H‑6b), 4.03‑4.07 (m, 1H, H‑5), 2.18, 2.17, 2.10, 2.05, 2.01 (5 × s, 15H, COC*H_3_* ); ^13^C-NMR (151 MHz, CDCl_3_) δ [ppm] = 170.8, 170.2, 169.9, 169.7, 168.2 (5x *C*OCH_3_), 90.7 (C-1), 70.7 (C-5), 68.8 (C-3), 68.4 (C-2), 65.6 (C-4), 62.2 (C-6), 21.0, 20.9, 20.9, 20.8, 20.8 (5 × CO*C*H_3_). The spectral data are in accordance with literature (2).

**Propargyl 2,3,4,6-tetra-*O*-acetyl-α-d-mannopyranoside 7**

1,2,3,4,6-Penta-*O*-acetyl-α,β-d-mannopyranose **6** (10.0 g, 25.6 mmol, 1 eq.) and propargyl alcohol (7.18 g, 7.48 mL, 128 mmol, 5 eq.) were dissolved in dichloromethane (100 mL). After stirring for 20 minutes at room temperature, the reaction mixture was cooled to 0 °C and BF_3_·OEt_2_ (16.22 mL, 128 mmol, 5 eq.) was added dropwise. The mixture was stirred for 15 min at this temperature, then at room temperature for 24 h. The solution was treated with saturated NaHCO_3_ solution (25 mL) the aqueous layer was extracted with dichloromethane (2 × 50 mL) and the combined organic layers were dried over anhydrous MgSO_4_. The solvent was removed *in vacuo* and the residue was purified by flash column chromatography (cyclohexane/ethyl acetate, 1:1) to give the title compound (7.23 g, 18.7 mmol, 73 %) as a colorless viscous oil. *R_f_* = 0.43 (silica gel, cyclohexane/ethyl acetate, 1:1);

^1^H-NMR (400 MHz, CDCl_3_) δ [ppm] = 5.36 (m, 3H, H‑2, H–3, H‑4), 5.02 (d, 1H, *^3^J* = 1.7 Hz, H‑1), 4.31–4.25 (m, 3H, H-6_a_, C*H*_2_–C≡C), 4.10 (dd, *^2^J* = 12.2 Hz, *^3^J* = 2.5 Hz, 1H, H‑6_b_), 4.01 (ddd, *^3^J* = 9.3 Hz, *^3^J* = 5.2 Hz, *^3^J* = 2.5 Hz, 1H, H‑5), 2.47 (t, *^4^J* = 2.4 Hz, 1H, C*H*), 2.15, 2.09, 2.03, 1.98 (4 × s, 12H, COC*H_3_* ); ^13^C-NMR (101 MHz, CDCl_3_) δ [ppm] = 170.8, 170.1, 170.0, 169.8 (4 × *C*OCH_3_), 96.4 (C-1), 86.0 (*C*≡CH), 75.7 (C≡*C*H), 69.5 (C-2), 69.1 (C-5), 69.1 (C-3), 66.1 (C-4), 62.4 (C-6), 55.1 (*C*H_2_), 21.0, 20.9, 20.8, 20.8 (4 × CO*C*H_3_). The spectral data are in accordance with literature (3).

**Propargyl α-d-mannopyranoside 8**

Propargyl 2,3,4,6-tetra-*O*-Acetyl-α-d-mannopyranoside **7** (7.00 g, 18.1 mmol) was dissolved in methanol (70 mL) and sodium methoxide was added until pH 9–10 (approx. 60 mg). The reaction mixture was stirred at room temperature for 16 h. Subsequently, the solution was neutralized by Amberlite^®^ 120 H^+^ resin until pH 7. The mixture was filtered over Celite^®^, which was washed thoroughly with methanol. The solvent was removed *in vacuo* to afford the desired 1-propargyl-α-d-mannopyranoside (3.50 g, 16.1 mmol, 89 %) as a highly viscous syrup, which solidified soon to an amorphous solid. *R_f_* = 0.85 (RP-silica gel, acetonitrile/water, 1:9);

^1^H-NMR (400 MHz, MeOD) δ [ppm] = 4.96 (d, *^3^J* = 1.7 Hz, 1H, H‑1), 4.27 (d, 1H, *^4^J* = 2.4 Hz, C*H_2_*), 3.84 (dd, *^2^J* = 11.8 Hz, *^3^J* = 2.3 Hz, 1H, H‑6_a_), 3.79 (dd, 1H, *^3^J* = 3.1 Hz, *^3^J* = 1.7 Hz, H‑2), 3.74‑3.58 (m, 3H, H‑3, H–4, H–6_b_), 3.54–3.47 (m, 1H, H–5), 2.86 (t, *^4^J* = 2.4 Hz, C*H*); ^13^C-NMR (101 MHz, MeOD) δ [ppm] =  99.8 (C‑1), 80.0 (*C*≡CH), 76.0 (C≡*C*H), 75.1 (C‑5), 72.5 (C‑3), 72.0 (C‑2), 68.5 (C–4), 62.8 (C–6), 54.8 (*C*H_2_-C≡CH). The spectral data are in accordance with literature (3).

**Propargyl 2,4-*O*-di-benzoyl-α-d-mannopyranoside 9 and propargyl 2,6-*O*-di-benzoyl-α-d-mannopyranoside 10**

Trimethyl orthobenzoate (3.1 mL, 18.1 mmol, 2.6 eq.) was added to a mixture of 1-propargyl-α-d-mannopyranoside (**8**, 1.5 g, 6.87 mmol, 1 eq.), trifluoroacetic acid (45 µL) and camphorsulfonic acid (75 mg) in acetonitrile (60 mL) at room temperature. The suspension was stirred for 2 h, it was obtained a clear colorless solution. The solvent was removed *in vacuo* and the residue was taken up in acetonitrile (45 mL). The solution was treated with 10 % aqueous trifluoroacetic acid (2.6 mL) at room temperature. After stirring for 1 h, the solvent was removed *in vacuo* and the residue was co-evaporated with toluene (4 × 50 mL). The crude product was purified by flash column chromatography (cyclohexane/ethyl acetate, gradient 0 % to 60 % ethyl acetate, Isolera™ Flash Purification System) to afford the two title compounds (1.61 g, 3.78 mmol, 55 %, 1-propargyl 2,4-*O*-di-benzoyl-α-d-mannopyranoside; 1.11 g, 2.60 mmol, 38 %, 1-propargyl 2,6-*O*-di-benzoyl-α-d-mannopyranoside) as colorless foams.

**Propargyl 2,4-di-*O*-Benzoyl-α-d-mannopyranoside 9**

*R_f_* = 0.48 (silica gel, cyclohexane/ethyl acetate, 2:1); ^1^H-NMR (400 MHz, CDCl_3_) δ [ppm] = 8.12 – 8.05 (m, 4H, H–2–Ar), 7.64 – 7.58 (m, 2H, H–4–Ar), 7.51 – 7.44 (m, 4H, H–3–Ar), 5.52 (pseudo-t, *^3^J* = 10.0 Hz, 1H, H–4), 5.45 (dd, *^3^J* = 3.5, *^3^J* = 1.7 Hz, 1H, H–2), 5.25 (d, *^3^J* = 1.7 Hz, 1H, H–1), 4.45 (dd, *^3^J* = 9.8, *^3^J* = 3.5 Hz, 1H, H–3), 4.33 (d, *^4^J* = 2.4 Hz, 2H, C*H_2_*), 4.00 (ddd, *^3^J* = 10.0, *^3^J* = 4.1, *^3^J* = 2.3 Hz, 1H, H–5), 3.82 (dd, *^2^J* = 12.6, *^3^J_H,H_* = 2.4 Hz, 1H, H–6_a_), 3.74 (dd, *^2^J* = 12.6, *^3^J* = 4.1 Hz, 1H. H–6_b_), 2.51 (t, *^4^J* = 2.4 Hz, 1H, C*H*); ^13^C-NMR (101 MHz, CDCl_3_) δ [ppm] =  167.4, 166.1 (2 × *C*O–Ar), 133.9, 133.8 (2 × C–4–Ar), 130.1 (2 × C–2–Ar, 2 × C–2’–Ar), 129.3, 129.1 (2 × C_q_), 128.8, 128.7 (C–3–Ar), 96.7 (C‑1, *^1^J_C,H_* = 173 Hz), 78.4 (*C*≡CH), 75.6 (C≡*C*H), 72.8 (C‑2), 71.2 (C‑5), 70.3 (C‑4), 68.7 (C–3), 61.5 (C‑6), 55.4 (*C*H_2_-C≡CH). ${[a]}_{D}^{22}$ – 11.9° (c = 1.00, CHCl_3_); HRMS (ESI): calculated for [C_23_H_22_O_8_ + Na]^+^: 449.1212, found: 449.1225.

**Propargyl 2,6-di-*O*-Benzoyl-α-d-mannopyranoside 10**

*R_f_* = 0.23 (silica gel, cyclohexane/ethyl acetate, 2:1); ^1^H-NMR (400 MHz, CDCl_3_) δ [ppm] = 8.14 – 8.09 (m, 2H, H–2–Ar^6^), 7.93 – 7.88 (m, 2H, H–2–Ar^2^), 7.65 – 7.59 (m, 1H, H–4–Ar^2^), 7.53 – 7.48 (m, 1H, H‑4‑Ar^6^), 7.48 – 7.42 (m, 2H, H–3–Ar^6^), 7.26 – 7.21 (m, 2H, H–3–Ar^2^), 5.41 (dd, *^3^J* = 3.4 Hz, *^3^J* = 1.7 Hz, 1H, H–2), 5.15 (d, *^3^J* = 1.7, 1H, H–1), 4.93–4.87 (m, 1H, H–6_a_), 4.51 (dd, *^2^J* = 12.2, *^3^J* = 1.8 Hz, 1H, H‑6_b_), 4.31 (d, *^4^J* = 2.4 Hz, 2H, C*H_2_*), 4.20 (dd, *^3^J* = 8.7, *^3^J* = 3.3, 1H, H–3), 4.22–3.89 (m, 2H, H–4, H‑5), 2.47 (t, *^4^J* = 2.4 Hz, 1H, C*H*); ^13^C-NMR (101 MHz, CDCl_3_) δ [ppm] =  167.5 (*C*O–Ar^6^), 166.1 (*C*O–Ar^2^), 133.5, 133.5 (2 × C–4–Ar), 130.0, 129.9 (2 × C–2–Ar), 129.7, 129.4 (2 × C_q_), 128.7, 128.8 (2 × C–3‑Ar), 96.9 (C–1, *^1^J_C,H_* = 173 Hz), 78.5 (*C*≡CH), 75.5 (C≡*C*H), 72.1 (C‑2), 72.1 (C‑4), 71.5 (C–5), 70.0 (C‑3), 67.8 (C–4), 63.5 (C–6), 55.1 (*C*H_2_-C≡CH). ${[a]}_{D}^{22}$ – 17.7° (c = 1.00, CHCl_3_); HRMS (ESI): calculated for [C_23_H_22_O_8_ + H]^+^: 427.1393, found: 427.1404.

**1,2,3,4,6-Penta-*O*-benzoyl-α,β-d-mannopyranose 11**

Benzoyl chloride (70 mL, 380 mmol, 6.8 eq.) was added dropwise to a stirred solution of d‑mannose **5** (10.0 g, 55.6 mmol, 1 eq.) and 4-(dimethylamino)-pyridine (cat., 30 mg) in pyridine (120 mL) under argon at 0 °C. The reaction mixture was stirred for 40 minutes at this temperature, then for 36 h at room temperature. The solvent was removed *in vacuo* and the residue was taken up in dichloromethane (400 mL) and water (100 mL), the organic layer was washed with brine (200 mL), sat. NaHCO_3_ solution (2 × 200 mL) and again with brine (100 mL). The separated organic layer was dried over anhydrous MgSO_4_ and the solvent was removed *in vacuo*. The residue was taken up in 100 mL boiling EtOH and then slowly cooled to room temperature. The precipitate was collected by filtration und dried *in vacuo* to give the title compound as a colorless powder (36.6 g, 52.3 mmol, 94 %, mixture of both anomers α /β 4.3:1). α–anomer: *R_f_* = 0.48 (silica gel, toluene/ethyl acetate, 19:1); β–anomer: *R_f_* = 0.36 (silica gel, toluene/ethyl acetate, 19:1);

Signals assignable to α-anomer **11**: ^1^H-NMR (400 MHz, CDCl_3_) δ [ppm] = 8.23–7.84 (m, 10H, H–Ar), 7.71–7.27 (m, 15H, H–Ar), 6.64 (d, *^3^J* = 2.0 Hz, 1H, H‑1), 6.29 (pseudo-t, 1H, *^3^J* = 10.2 Hz H‑4), 6.08 (dd, *^3^J* = 10.3 Hz, *^3^J* = 3.3 Hz, 1H, H‑3), 5.92 (dd, *^3^J* = 3.3 Hz, *^3^J* = 2.0 Hz, 1H, H‑2), 4.70 (dd, *^3^J* = 12.2 Hz, *^3^J* = 3.7 Hz, 1H, H‑6_a_), 4.51 (pseudo-dt, *^3^J* = 10.0 Hz, *^3^J* = 2.9 Hz, 1H, H‑5), 4.51 (dd, *^3^J* = 12.2 Hz, *^3^J* = 3.7 Hz, 1H, H‑6_b_); ^13^C-NMR (101 MHz, CDCl_3_) δ [ppm] = 166.2, 165.8, 165.4, 165.3, 163.7 (5 × *C*O–Ar), 134.2, 133.8, 133.7, 133.5, 133.2 (5 × C_q_), 130.3, 130.1, 130.0, 129.9, 129.1, 129.0, 128.9, 128.8, 128.6, 128.6, 128.5 (C–Ar), 91.5 (*^1^J_CH_* = 180 Hz, C–1), 71.3 (C-5), 70.1 (C-3), 69.6 (C-2), 66.3 (C-4), 62.5 (C-6).

Signals assignable to β-anomer **11a**: ^1^H-NMR (400 MHz, CDCl_3_) δ [ppm] = 8.18–7.84 (m, 10H, H–Ar), 7.67–7.27 (m, 15H, H–Ar), 6.44 (d, *^3^J* = 1.2 Hz, 1H, H‑1), 6.18 (pseudo-t,  *^3^J* = 9.8 Hz, 1H, H‑4), 6.11 (dd, *^3^J* = 3.2 Hz, *^3^J* = 1.1 Hz, 1H, H‑2), 5.81 (dd, *^3^J* = 10.0 Hz, *^3^J* = 3.2 Hz, 1H, H‑3), 4.76 (dd, *^3^J* = 12.4 Hz, *^3^J* = 2.8 Hz, 1H, H‑6_a_), 4.56 (dd, *^3^J* = 12.3 Hz, *^3^J* = 4.4 Hz, 1H, H‑6_b_), 4.38 (ddd, *^3^J* = 9.7 Hz, *^3^J* = 4.3 Hz, *^3^J* = 2.8 Hz, 1H, H‑5); ^13^C-NMR (101 MHz, CDCl_3_) δ [ppm] = 166.2, 165.7, 165.6, 165.4, 164.3 (5 × *C*O–Ar), 133.9, 133.7, 133.7, 133.6, 133.2 (5 x C_q_), 130.3, 130.2, 130.0, 130.0, 129.5, 128.9, 128.8, 128.8, 128.6, 128.6, 128.6, 128.5 (C–Ar), 91.4 (*^1^J_CH_* = 163 Hz, C–1), 73.5 (C-5), 71.7 (C-3), 69.5 (C-2), 66.5 (C-4), 62.8 (C-6). The spectral data are in accordance with literature (4).

**2,3,4,6-Tetra-*O*-benzoyl-α-d-mannopyranosyl bromide 12**

1,2,3,4,6-Penta-*O*-benzoyl-α,β-d-mannopyranose **11** (5 g, 7.14 mmol, 1 eq.) was dissolved in dichloromethane (25 mL) and treated with HBr (33 % in acetic acid, 12.3 mL, 71.4 mmol, 10 eq.) at 0 °C. After 2 h, another portion of HBr (1 mL, 33 % in acetic acid) was added, TLC (cyclohexane/ethyl acetate 2:1) showed complete conversion after 4 h. Then, the reaction mixture was diluted with dichloromethane (25 mL), washed with water (25 mL), saturated aqueous NaHCO_3_ solution (4 × 25 mL) and brine (25 mL). The separated organic layer was dried over anhydrous MgSO_4_ and the solvent was removed *in vacuo* to give the title compound (4.51 g, 6.85 mmol, 94 %,) as a yellowish viscous oil. *R_f_* = 0.60 (silica gel, cyclohexane/ethyl acetate, 2:1);

^1^H-NMR (400 MHz, CDCl_3_) δ [ppm] = 8.13–7.82 (m, 8H, H–Ar), 7.64–7.27 (m, 12H, H–Ar), 6.59 (d, *^3^J* = 1.1 Hz, 1H, H‑1), 6.32–6.22 (m, 2H, H‑3, H‑4), 5.91 (dd, *^3^J* = 2.9 Hz, *^3^J* = 1.7 Hz, 1H, H‑2), 4.75 (dd, *^2^J* = 12.5 Hz, *^3^J* = 2.4 Hz, 1H, H‑6_a_), 4.66 (pseudo-dt, *^3^J* = 9.4 Hz, *^3^J* = 3.1 Hz, 1H, H‑5), 4.51 (dd, *^2^J* = 12.5 Hz, *^3^J* = 3.7 Hz, 1H, H‑6_b_); ^13^C-NMR (101 MHz, CDCl_3_) δ [ppm] = 166.1, 165.5, 165.4, 165.1 (4 x *C*O–Ar), 133.9, 133.8, 133.5, 133.3 (4 x C_q_), 130.0, 129.9, 129.9, 129.8, 128.9, 128.8, 128.8, 128.7, 128.6, 128.5 (C–Ar), 83.4 (C–1), 73.3 (C-5), 73.1 (C-2), 69.2 (C-3), 66.1 (C-4), 61.9 (C-6). The spectral data are in accordance with literature (5).

**2,4-Di-*O*-benzoyl-3,6-di-*O*-(2,3,4,6-tetra-*O*-benzoyl-α-d-mannopyranoside)-1-propargyl-α-d-mannopyranoside 13**

1-Propargyl 2,4-*O*-di-benzoyl-α-d-mannopyranoside **9** (415 mg, 0.97 mmol, 1 eq.) and 2,3,4,6-tetra-*O*-benzoyl-α-d-mannosylpyranosyl bromide **12** (1.27 g, 1.94 mmol, 2 eq.) was dissolved in dichloromethane (11 mL) in a thoroughly flame dried flask under argon. The solution was stirred for 20 min at 0 °C. AgOTf (543 mg, 2.11 mmol, 2.4 eq.) was dissolved in toluene (3 mL) and added dropwise to the solution. Immediately a yellowish precipitate is formed and after 1.5 h the greenish reaction mixture was treated with NEt_3_ (500 µL). It was filtered over Celite^®^, which was washed thoroughly with dichloromethane. The solvent was removed *in vacuo* and the residue was purified by flash column chromatography (cyclohexane/ethyl acetate, gradient 0 % to 50 % ethyl acetate, Isolera™ Flash Purification System) to afford the title compound (1.06 g, 0.67 mmol, 69 %) as a colorless foam. *R_f_* = 0.40 (silica gel, cyclohexane/ethyl acetate, 2:1);

^1^H-NMR (600 MHz, CDCl_3_) δ [ppm] = 8.33–8.30 (m, 2H, H–Ar), 8.14–8.10 (m, 4H, H–Ar), 8.09–8.01 (m, 6H, H–Ar), 7.88–7.80 (m, 6H, H–Ar), 7.74–7.70 (m, 2H, H–Ar), 7.64–7.47 (m, 8H, H–Ar), 7.46–7.27 (m, 20H, H–Ar), 7.22–7.19 (m, 2H, H–Ar), 6.14 (t, *^3^J* = 10.1 Hz, 1H, H–4‘‘), 6.02–5.96 (m, 2H, H–3‘‘, H–4‘), 5.93 (t, *^3^J* = 10.0 Hz, 1H, H–4), 5.79–5.76 (m, 2H, H–2‘‘, H–2), 5.71 (dd, *^3^J* = 10.1 Hz, *^3^J* = 2.8 Hz, 1H, H–3‘), 5.37–5.35 (m, 3H, H–1, H–1‘, H–2‘), 5.15 (d, *^3^J* = 1.8 Hz, 1H, H–1‘‘), 4.68 (dd, *^3^J* = 9.8 Hz, *^3^J* = 3.5 Hz, 1H, H–3), 4.65–4.52 (m, 4H, H–6_a_‘‘, H-5‘‘, H–5‘, H–6_a_‘), 4.47 (dd, *^2^J* = 16.0 Hz, *^4^J* = 2.4 Hz, 1H, O–C*H*_2,a_–C$\equiv$C), 4.43–4.36 (m, 3H, H–6_b_‘‘, H–6_b_‘, O–C*H*_2,b_–C$\equiv$C), 4.33 (ddd, *^3^J* = 10.3 Hz, *^3^J* = 6.1 Hz, *^4^J* = 2.1 Hz, 1H, H–5), 4.17 (dd, *^2^J* = 10.8 Hz, *^3^J* = 6.2 Hz, 1H, H–6_a_ ), 3.81 (dd, *^2^J* = 10.9 Hz, *^3^J* = 2.1 Hz, 1H, H–6_b_), 2.60 (t, *^4^J* = 2.4 Hz, 1H, –C$\equiv$C–*H*); ^13^C-NMR (151 MHz, CDCl_3_) δ [ppm] = 166.7, 166.3, 166.2, 165.7, 165.6, 165.4, 165.4, 165.4, 164.8, 164.7 (10 × *C*O), 133.8, 133.6, 133.6, 133.5, 133.4, 133.4, 133.2, 133.1, 133.1, 133.0 (10 × C–4–Ar), 130.4, 130.1, 130.1, 130.0, 130.0, 129.8, 129.8, 129.8, 129.7 (C–Ar), 129.4, 129.3, 129.3, 129.3, 129.3, 129.1, 129.1, 129.1, 129.0, 129.0 (10 × C_q_–Ar), 128.7, 128.6, 128.5, 128.4, 128.4, 128.3 (C–Ar), 99.6 (C–1‘), 97.6 (C–1‘‘), 96.4 (C–1), 78.3 (–*C*$\equiv$C–H), 76.1 (‑C$\equiv$*C*–H), 76.0 (C–3), 71.7 (C–2‘‘), 70.4 (C–3‘‘), 70.4 (C–2), 70.3 (C–5), 70.3 (C–2‘), 69.7 (C–3‘), 69.4 (C–5‘), 69.0 (C–5‘‘), 68.6 (C–4), 67.1(C–6), 66.7 (C–4‘), 66.7 (C–4‘‘), 62.8 (C–6‘‘), 55.2 (–*C*H_2_–C$\equiv$C). HRMS (ESI): calculated for [C_91_H_74_O_26_ + Na]^+^: 1605.4366, found: 1605.4390.

**1-Methanesulfonyl-2-(2-(2-(2-methanesulfonylethoxy)ethoxy)ethoxy)ethane 15**

Methanesulfonyl chloride (4.48 mL, 56.6 mmol, 2.2 eq.) and tetraethylene glycol **14** (5.00 g, 25.7 mmol, 1 eq.) were dissolved in dichloromethane (125 mL) at 0 °C. The reaction mixture was treated with NEt_3_ (10.7 mL, 77.2 mmol, 3 eq.) and stirred for 45 min. Subsequently water (125 mL) was added and the separated organic layer was washed with ice cold 2 *N* HCl (125 mL), saturated aqueous NaHCO_3_ solution (125 mL) and brine (125 mL). The organic layer was dried over anhydrous Na_2_SO_4_ and the solvent was removed *in vacuo* to give the title compound (8.78 g, 25.1 mmol, 98 %) as a yellowish oil. *R_f_* = 0.20 (silica gel, cyclohexane/ethyl acetate, 1:2);

^1^H-NMR (300 MHz, CDCl_3_) δ [ppm] = 4.40–3.35 (m, 4H, 2 × MsO–C*H_2_*), 3.79–3.74 (m, 4H, MsO‑CH_2_‑C*H_2_*), 3.70–3.61 (m, 8H, MsO–Et-O[(C*H_2_*)_2_–O–]_2_–Et–OMs), 3.07 (m, 6H, 2 × –CH_3_); ^13^C-NMR (75 MHz, CDCl_3_) δ [ppm] = 70.8 (2 × CH_2_–*C*H_2_–O), 70.7 (2 × *C*H_2_–CH_2_–O), 69.3 (2 × MsO–*C*H_2_), 69.2 (2 × MsO–CH_2_–*C*H_2_), 37.8 (2 × –*C*H_3_). The spectral data are in accordance with literature (6).

**1-Azido-2-(2-(2-(2-azidoethoxy)ethoxy)ethoxy)ethane 16**

1-Methanesulfonyl-2-(2-(2-(2-methanesulfonylethoxy)ethoxy)ethoxy)ethane **15** (2.00 g, 5.71 mmol, 1 eq.) was dissolved in a mixture of EtOH (12 mL) and dimethylacetamide (3 mL) and NaN_3_ (1.15 g, 17.7 mmol, 3.1 eq.) was added. The reaction mixture was refluxed for 6 h and subsequently poured slowly into water (20 mL) and dichloromethane (20 mL). The separated organic layer was washed with water (50 mL) and brine (50 mL). The organic layer was dried over anhydrous Na_2_SO_4_ and the solvent was removed *in vacuo*. The residue was co-evaporated with toluene (4 × 50 mL) to give the title compound (1.36 g, 5.57 mmol, 98 %) as a colorless oil. *R_f_* = 0.60 (silica gel, cyclohexane/EtOAc, 4:2);

^1^H-NMR (300 MHz, CDCl_3_) δ [ppm] = 3.72–3.62 (m, 12H, 2 × N_3_–CH_2_–C*H_2_*, 2 × C*H_2_*–C*H_2_*–O), 3.39 (t, *^3^J* = 5.1 Hz, 4H, N_3_–C*H_2_*); ^13^C-NMR (75 MHz, CDCl_3_) δ [ppm] = 70.9 (2 × *C*H_2_–*C*H_2_–O), 70.2 (2 × N_3_‑CH_2_–*C*H_2_), 50.8 (2 × N_3_–*C*H_2_). The spectral data are in accordance with literature (7).

**(1-(2-(2-(2-(2-Azidoethoxy)ethoxy)ethoxy)ethyl)-1*H*-1,2,3-triazole-4-yl)methoxy)-2,4-di-*O*-benzoyl-3,6-di-*O*-(2,3,4,6-tetra-*O*-benzoyl-α-d-mannopyranosyl)-α-d-mannopyranoside 17**

Dimethylformamide (50 mL) was degassed by freeze-pump-thaw-cycle three times then 2,4-di-*O*-benzoyl-3,6-di-*O*-(2,3,4,6-tetra-*O*-benzoyl-α-d-mannopyranoside)-1-propargyl-α-d-mannopyranoside **13** (325 mg, 0.21 mmol, 1 eq.) and 1-azido-2-(2-(2-(2-azidoethoxy)ethoxy)ethoxy)ethane **16** (725 mg, 3.08 mmol, 15 eq.) was dissolved under argon atmosphere. Subsequently, the freeze-pump-thaw-cycle was repeated again for three times. CuBr (15 mg, 0.10 mmol, 50 mol %) and *N*,*N*,*N*′,*N*′,*N*′′-pentamethyldiethylenetriamine (50 µL, 0.24 mmol, 85 mol %) was added and the teal reaction mixture was stirred at 45 °C for 1.5 h. Subsequently, it was diluted with ethyl acetate (40 mL) and washed with aqueous saturated NH_4_Cl solution (2 × 40 mL). The aqueous layer was diluted with water so that all salts were dissolved and washed with ethyl acetate (30 mL). The combined organic layers were washed with brine and dried over anhydrous MgSO_4_. The solvent was removed *in vacuo* and the residue was purified by flash column chromatography (cyclohexane/ethyl acetate, gradient 0 % to 90 % ethyl acetate, Isolera™ Flash Purification System) to afford the title compound (302 mg, 0.40 mmol, 81 %) as a colorless oil. *R_f_* = 0.28 (silica gel, cyclohexane/ethyl acetate, 2:4);

^1^H-NMR (600 MHz, CDCl_3_) δ [ppm] = 8.33–8.29 (m, 2H, H–Ar), 8.14–8.00 (m, 10H, H–Ar), 7.89 (s, 1H, H_triazole_), 7.86–7.82 (m, 4H, H–Ar), 7.78–7.75 (m, 2H, H–Ar), 7.72–7.69 (m, 2H, H–Ar), 7.61–7.35 (m, 20H, H–Ar), 7.31–7.27 (m, 8H, H–Ar), 7.22–7.18 (m, 2H, H–Ar), 6.14 (t, *^3^J* = 10.1 Hz, 1H, H–4‘‘), 6.06‑5.99 (m, 3H, H–3‘‘, H–4‘, H–4), 5.82–5.81 (m, 1H, H–2‘‘), 5.76–5.74 (m, 1H, H–2), 5.70 (dd, *^3^J* = 10.0 Hz, *^3^J* = 2.8 Hz, 1H, H–3‘), 5.34–5.32 (m, 2H, H–1‘, H–2‘), 5.27 (s, 1H, H–1), 5.19 (s, 1H, H–1‘‘), 5.00 (d, *^2^J* = 12.2 Hz, 1H, O–C*H_2a_–*C=C), 4.84 (d, *^2^J* = 12.2 Hz, 1H, O–C*H_2b_–*C=C), 4.64 (dd, *^3^J* = 9.8 Hz, *^3^J* = 3.4 Hz , 1H, H–3), 4.61 (dd, *^2^J* = 12.4 Hz, *^3^J* = 2.5 Hz, 1H, H–6_a_‘), 4.58–4.53 (m, 4H, triazole–C*H_2_*, H–6a‘‘, H–5‘‘), 4.47 (dt, *^3^J* = 10.1 Hz, *^3^J* = 2.9 Hz, 1H, H–5‘), 4.42–4.39 (m, 1H, H–5), 4.34 (dd, *^2^J* = 12.2 Hz, *^3^J* = 4.4 Hz, 1H, H–6_b_‘‘), 4.29 (dd, *^2^J* = 12.4 Hz, *^3^J* = 3.1 Hz, 1H, H–6_b_‘), 4.18 (dd, *^2^J* = 10.9 Hz, *^3^J* = 5.2 Hz, 1H, H–6_a_), 3.89 (t, *^3^J* = 5.0 Hz, 2H, triazole–CH_2_–C*H_2_*), 3.80 (dd, *^2^J* = 11.0 Hz, *^3^J* = 2.2 Hz, 1H, H–6_b_), 3.64 (m, 10H, alkyl C*H_2_*), 3.33 (t, *^3^J* = 5.0 Hz, 2H, N_3_–C*H_2_*); ^13^C-NMR (151 MHz, CDCl_3_) δ [ppm] = 166.3, 166.2, 166.2, 165.7, 165.6, 165.3, 165.3, 165.3, 164.8, 164.7 (10 × *C*O), 143.3 (C_q_–in triazole), 133.7, 133.5, 133.5, 133.5, 133.4, 133.4, 133.2, 133.1, 133.1, 133.0 (10 × C–4–Ar), 130.3, 130.1, 130.1, 130.0, 130.0, 129.9, 129.9, 129.9, 129.8, 129.7, 129.7 (C–Ar), 129.4, 129.4, 129.3, 129.3, 129.3, 129.1, 129.1, 129.1, 129.0, 129.0 (10 × C_q_–Ar), 128.6, 128.6, 128.6, 128.5, 128.5, 128.5, 128.5, 128.4, 128.4 128.2 (C–Ar), 124.6 (triazole-C*H*), 99.7 (C–1‘), 97.8 (C–1‘‘), 97.0 (C–1), 76.9 (C–3), 71.8 (C–2), 70.7 (3 signals, respectively alkyl *C*H_2_), 70.4 (C–2‘), 70.3 (C–3‘‘), 70.3 (C–2‘‘), 70.1 (alkyl *C*H_2_), 69.9 (C–5), 69.6 (C–5‘), 69.5 (triazole–CH_2_–*C*H_2_), 69.5 (C–3‘), 69.0 (C–5‘‘), 68.4 (C–4), 66.9 (C–6), 66.6 (C–4‘), 66.5 (C–4‘‘), 62.8 (C–6‘‘), 62.5 (C–6‘), 61.0 (O–*C*H_2_*–*C=C), 50.7 (N_3_–*C*H_2_), 50.3 (triazole–*C*H_2_). HRMS (ESI): calculated for [C_99_H_90_N_6_O_29_ + Na]^+^: 1849.5650, found: 1849.5684.

**(1-(2-(2-(2-(2-Azidoethoxy)ethoxy)ethoxy)ethyl)-1*H*-1,2,3-triazole-4-yl)methoxy)-3,6-di-*O*-α-d-mannopyranosyl-α-d-mannopyranoside 18**

(1-(2-(2-(2-(2-Azidoethoxy)ethoxy)ethoxy)ethyl)-1*H*-1,2,3-triazole-4-yl)methoxy)-2,4-di-*O*-benzoyl-3,6-di-*O*-(2,3,4,6-tetra-*O*-benzoyl-α-d-mannopyranosyl)-α-d-mannopyranoside **17** (500 mg, 0.27 mmol, 1 eq.) was dissolved in a mixture of MeOH (4 mL) and dichloromethane (4 mL) and treated with NaOMe (15 mg, 0.27 mmol,1 eq.). After stirring for 24 hours at room temperature, RP-TLC (acetonitrile/water 2:8) showed complete conversion. The reaction mixture was neutralized with conc. HCl (two drops were required). Subsequently the solvents were removed *in vacuo* and the residue was co-evaporated with toluene (3 × 50 mL). The residue was purified by preparative HPLC (MeOH/H_2_O, 80:20 for 10 min, then 50:50 for 10 min, 37.5 mL/min, R_t_ 14.7 min) to give the desired unprotected product (150 mg, 0.19 mmol, 71 %) as a colorless lyophylisat. *R_f_* = 0.57 (RP-silica gel, acetonitrile/water, 2:8);

^1^H-NMR (600 MHz, D_2_O) δ [ppm] = 8.07 (s, 1H, H–Ar), 5.03 (d, *^3^J* = 1.7 Hz, 1H, H–1‘), 4.89 (d, *^3^J* = 1.7 Hz, 1H, H–1), 4.85 (d, *^3^J* = 1.7 Hz, 1H, H–1‘‘), 4.79–4.78 (m, 1H, O–C*H_2a_–*C=C), 4.69 (d, *^2^J* = 12.6 Hz, 1H, O–C*H_2b_–*C=C), 4.60 (t, *^3^J* = 5.0 Hz, 2H, triazole–C*H_2_*), 4.55 (dd, *^3^J* = 3.0 Hz, *^3^J* = 1.7 Hz, 1H, H–2), 4.01 (dd, *^3^J* = 3.3 Hz, *^3^J* = 1.7 Hz, 1H, H–2‘), 3.90–3.56 (m, 26H, all other), 3.45–3.42 (m, 2H, N_3_–C*H_2_*); ^13^C-NMR (151 MHz, D_2_O) δ [ppm] = 143.4 (C_q_–triazole), 125.4 (*C*H in triazole), 102.3 (C–1‘), 99.6 (C–1), 99.3 (C–1‘‘), 78.5 (C–3), 73.2, 72.6, 71.2, 70.5, 70.3, 69.9 (C–2‘), 69.8, 69.6 (C–2), 69.5, 69.4, 69.1, 68.7 (triazole–CH_2_–*C*H_2_), 66.6, 65.4, 64.9 (C–6), 60.9, 59.9, 50.0, 50.0 (N_3_–*C*H_2_, triazole–*C*H_2_). ${[a]}_{D}^{22}$ – 63.2° (c = 0.75, MeOH); HRMS (ESI): calculated for [C_29_H_50_N_6_O_19_ + Na]^+^: 809.3028, found: 809.3030.

**(1-(2-(2-(2-(2-azidoethoxy)ethoxy)ethoxy)ethyl)-1*H*-1,2,3-triazol-4-yl)methoxy-2,3,4,6-tetra-*O*-acetyl-α-d-mannopyranoside 19**

1-Propargyl-2,3,4,6-tetra-*O*-acetyl-α-d-mannopyranoside **7** (250 mg, 0.65 mmol, 1 eq.) was dissolved in a mixture of tetrahydrofuran (5 mL) and water (5 mL). 1-azido-2-(2-(2-(2-azidoethoxy)ethoxy)ethoxy)ethane **16** (400 mg, 1.55 mmol, 2.4 eq.), CuCO_4_ (21 mg, 0,13 mmol, 20 mol %) and sodium ascorbate (40 mg, 0.20 mmol, 30 mol %) was added successively. The reaction mixture was stirred for four days at room temperature and subsequently diluted with ethyl acetate (30 mL). The mixture was washed with saturated aqueous NH_4_Cl solution (2x 30 mL) and the organic layer was washed with brine (30 mL). The organic layer was washed with brine and dried over anhydrous MgSO_4_. The solvent was removed *in vacuo* and the residue was purified by flash column chromatography (dichloromethane/methanol, isocratic 1 % methanol, then 3 % methanol, Isolera™ Flash Purification System) to afford the title compound (175 mg, 0.28 mmol, 43 %) as a colorless oil. The di-clicked compound 1,1´-[oxybis(ethane-2,1-diyloxyethane-2,1-diyl)]bis(4-methoxy-2,3,4,6-tetra-*O*-acetyl-α-d-mannopyranoside)-1*H*-1,2,3-triazole (85 mg, 0.084 mmol, 13 %) was obtained as a side product. *R_f_* = 0.42 (silica gel, dichloromethane/methanol, 95:5);

^1^H-NMR (400 MHz, CDCl_3_) δ [ppm] = 7.79 (s, 1H, H_Triazole_), 5.33–5.28 (m, 2H, H–3, H–4), 5.23 (dd, *^3^J* = 3.1, *^3^J* = 1.8 Hz, 1H, H–2), 4.97 (d, *^3^J* = 1.7 Hz, 1H, H–1), 4.84 (d, *^2^J* = 12.2 Hz, 1H, O–C*H_2a_–*C=C), 4.68 (d, *^2^J* = 12.2 Hz, 1H, O–C*H_2b_–*C=C), 4.56 (t, *^3^J* = 5.0 Hz, 2H, Triazole–C*H_2_*), 4.30 (dd, *^2^J* = 12.3, *^3^J* = 5.1 Hz, 1H, H–6_a_), 4.14–4.05 (m, 2H, H–6_b_, H–5), 3.89 (t, *^3^J* = 5.0 Hz, 2H, Triazole–CH_2_–C*H_2_*), 3.70 – 3.61 (m, 10H, Alkyl C*H_2_*), 3.38 (t, *^3^J* = 5.0 Hz, 2H, N_3_–C*H_2_*), 2.15 (s, 3H, –COC*H*_3_), 2.12 (s, 3H, –COC*H*_3_), 2.03 (s, 3H, –COC*H*_3_), 1.97 (s, 3H, –COC*H*_3_); ^13^C-NMR (101 MHz, CDCl_3_) δ [ppm] =  170.7, 170.0, 169.9, 169.7 (4x –*C*OCH_3_), 143.3 (*C*_q_–Triazole), 124.3 (*C*H–Triazole), 96.8 (C–1), 70.7, 70.6, 70.6, 70.1 (Alkyl–C), 69.4, 69.5 (C–2, Triazole–CH_2_–*C*H_2_), 69.1 (C–3), 68.7 (C–5), 66.1 (C–4), 62.4 (C–6), 61.0 (*C*H_2_–C=CH), 50.7 (N_3_–*C*H_2_), 50.3 (Triazole–*C*H_2_), 20.9, 20.8, 20.7, 20.7 (4x –CO*C*H_3_). ${[a]}_{D}^{22}$ + 24.0° (c = 0.10, CHCl_3_); HRMS (ESI): calculated for [C_25_H_38_N_6_O_13_ + Na]^+^: 653.2395, found: 653.2402.

**(1-(2-(2-(2-(2-azidoethoxy)ethoxy)ethoxy)ethyl)-1*H*-1,2,3-triazol-4-yl)methoxy-α-d-mannopyranoside 20**

(1-(2-(2-(2-(2-azidoethoxy)ethoxy)ethoxy)ethyl)-1*H*-1,2,3-triazol-4-yl)methoxy-2,3,4,6-tetra-*O*-acetyl-α-d-mannopyranoside **19** (100 mg, 0.16 mmol) was dissolved in MeOH (4 mL) and NaOMe was added until pH 9–10 (approx. 10 mg). The reaction mixture was stirred at room temperature for 17 hours. Subsequently, the solution was neutralized by Amberlite^®^ 120 H^+^ resin until pH 7. The mixture was filtered over Celite^®^, which was washed thoroughly with methanol. The solvent was removed *in vacuo* and the residue was purified by preparative HPLC (MeCN/H_2_O, gradient 5 % to 60 % in 20 min, R_t_ 7.49 min) to give the desired unprotected product as an amorphous solid (54 mg, 0.12 mmol, 80 %). *R_f_* = 0.49 (silica gel, ethyl acetate/methanol, 1:1);

^1^H-NMR (400 MHz, CD_3_OD) δ [ppm] = 8.07 (s, 1H, H–Ar), 4.86 (d, *^3^J* = 1.7 Hz, 1H, H‑1), 4.80 (d, *^2^J* = 12.4 Hz, 1H, O–C*H_2a_–*C=C), 4.65 (d, *^2^J* = 12.4 Hz, 1H, O–C*H_2b_–*C=C), 4.59 (t, *^3^J* = 5.0 Hz, 2H, triazole–C*H_2_*), 3.90 (t, *^3^J* = 5.0 Hz, 2H, triazole–CH_2_–C*H_2_*), 3.85 (dd, *^2^J* = 11.8 Hz, *^3^J* = 2.0 Hz, 1H, H–6_a_), 3.78 (dd, *^3^J* = 3.2 Hz, *^3^J* = 1.7 Hz, 1H, H–2), 3.72 (dd, *^2^J* = 11.8 Hz, *^3^J* = 5.6 Hz, 1H, H–6_b_), 3.67–3.56 (m, 13H, H–3, H–4, H–5, 5x alkyl CH_2_), 3.37 (t, *^3^J* = 4.9 Hz, 2H, N_3_–C*H*_2_); ^13^C-NMR (151 MHz, CD_3_OD) δ [ppm] = 145.1 (C_q_–triazole), 126.2(*C*H in triazole), 102.3 (C–1‘), 100.8 (C–1), 75.0 (C–5), 72.5 (C–3), 70.0 (C–2), 71.6, 71.6, 71.5, 70.4, 70.1 (5x alkyl *C*H_2_), 70.3 (triazole–CH_2_–*C*H_2_), 68.6 (C–4), 63.0 (C‑6), 60.7 (O–*C*H_2_–C=C) (triazole–CH_2_–*C*H_2_), 51.8 (N_3_–*C*H_2_), 51.5 (triazole–*C*H_2_). ${[a]}_{D}^{22}$ + 24.0° (c = 0.10, MeOH); HRMS (ESI): calc. for [C_17_H_30_N_6_O_9_ + Na]^+^: 485.1972, found: 485.1950.

## Spectra obtained for azide-modified saccharides
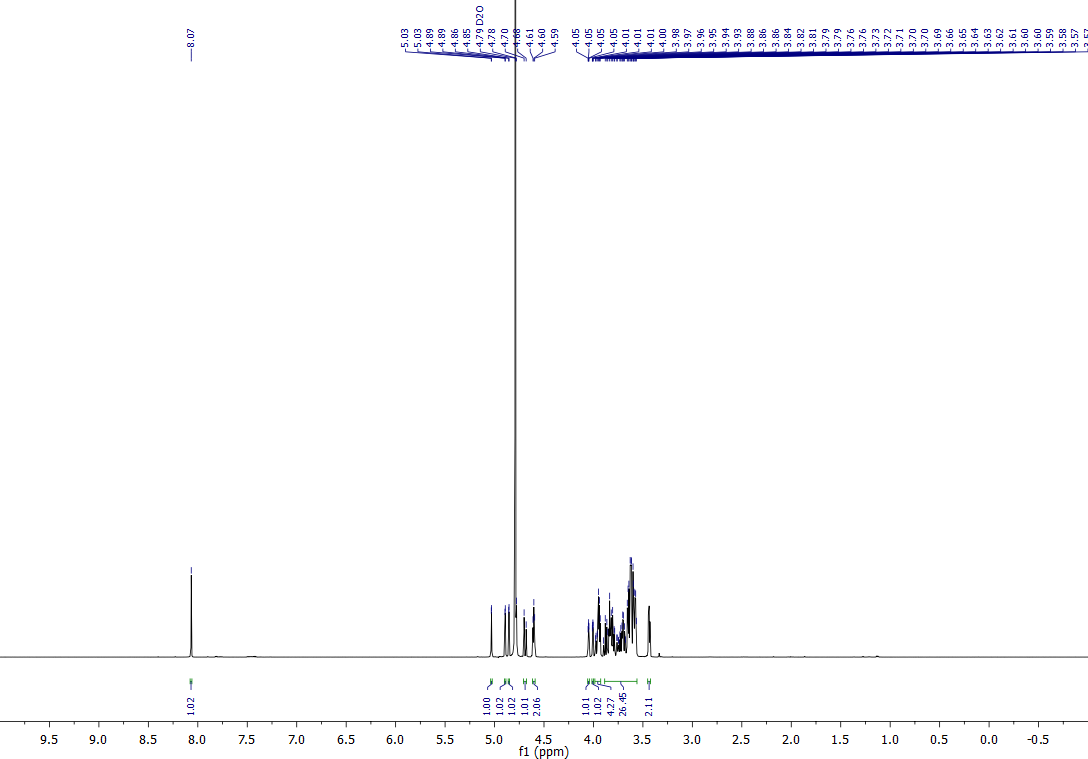


Supplementary Figure 10. ^1^H-NMR (600 MHz, D_2_O), compound 18.

**
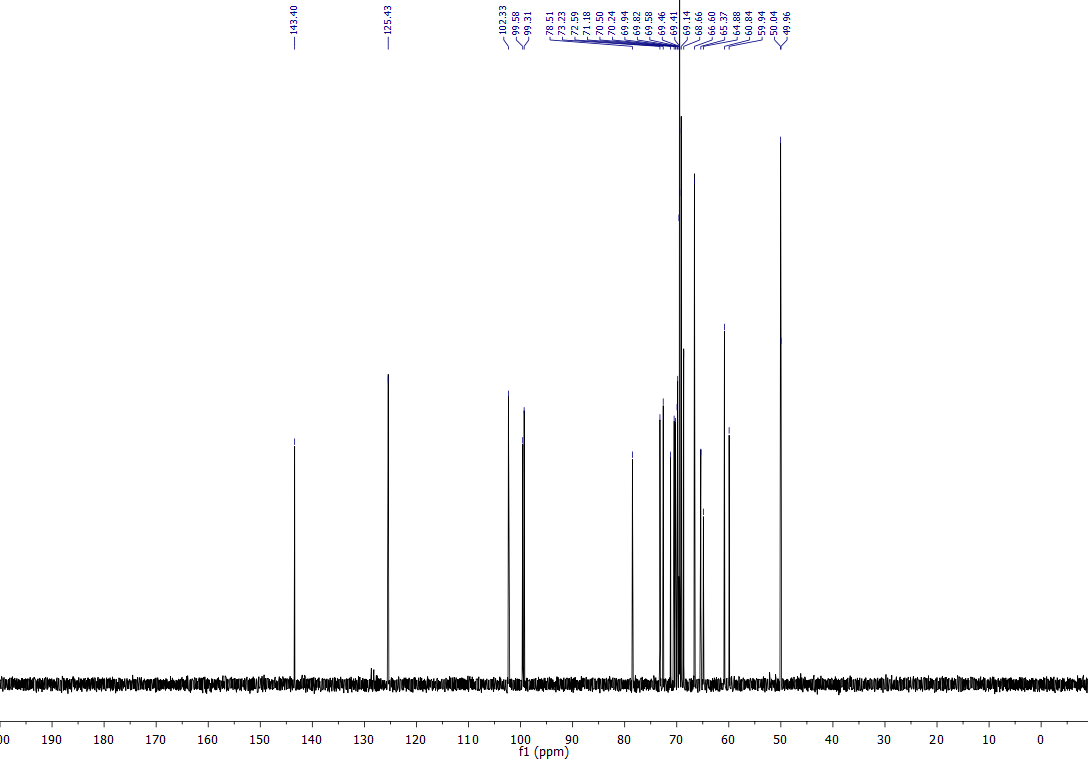
**

Supplementary Figure 11. ^13^C-NMR (150 MHz, D_2_O), compound 18.

**
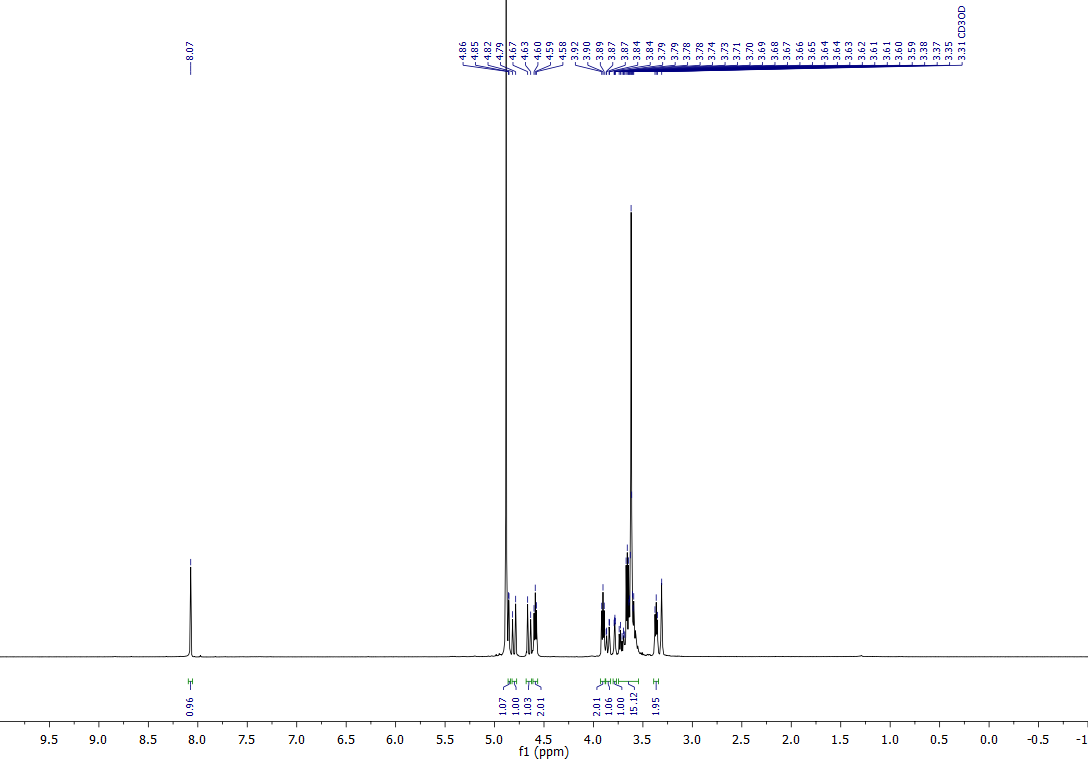
**

Supplementary Figure 12. ^1^H-NMR (400 MHz, CD_3_OD), compound 20.

**
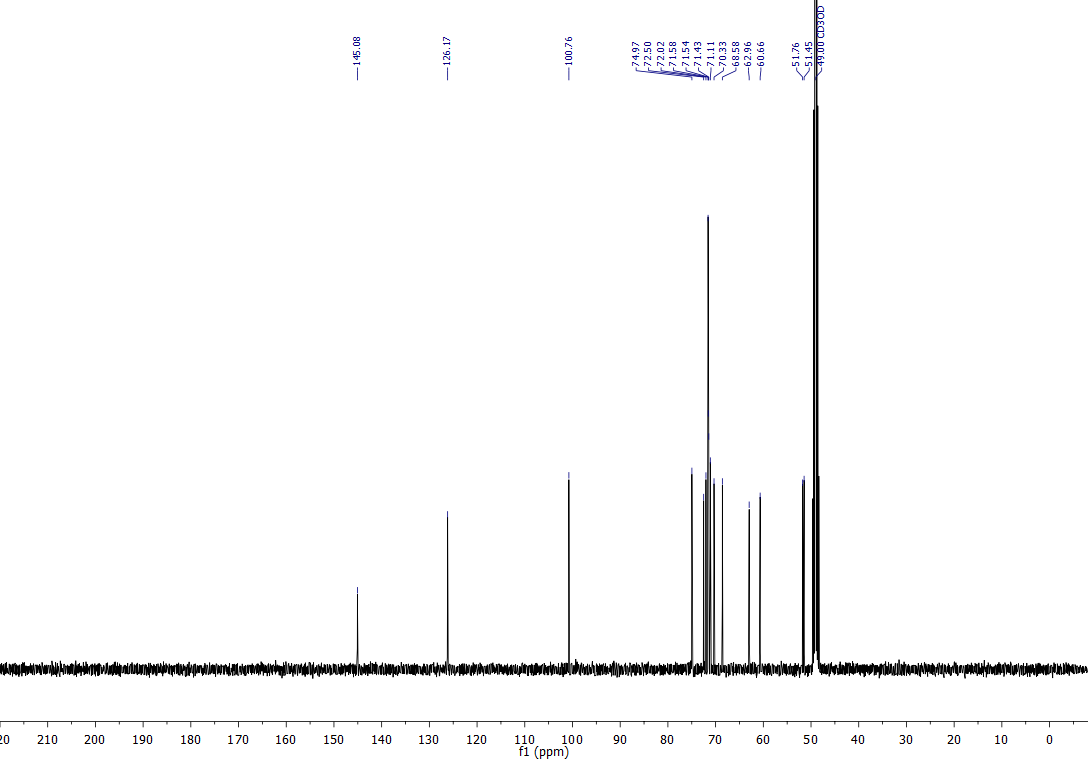
**

Supplementary Figure 13. ^13^C-NMR (100 MHz, CD_3_OD), compound 20.

# Synthesis of azide-modified perylene dye

***N*-(2,6-Diisopropylphenyl)-*N‘*-(4-carboxyethyl)-1,6,7,12-tetra(4-sulfophenoxy)-perylen-3,4:9,10-tetracarboxydiimid (23)**

*N*-(2,6-Diisopropylphenyl)-*N‘*-(4-aminoethyl)-1,6,7,12-tetra(4-sulfophenoxy)-perylen-3,4:9,10-tetra-carboxydiimid (8) **21** (50 mg, 0.04 mmol, 1.0 eq. ) was dissolved in DMF (5 mL) and adjusted to a basic pH of 11.2 with DIPEA (326 mg, 2.55 mmol). Subsequently, a solution of azidoacetic acid-NHS-ester **22** (13 mg, 0.06 mmol, 1.5 eq.) in DMF (2 mL) was added to the mixture. The reaction was stirred for 30 minutes at room temperature. Solvents were removed *in* *vacuo* and the solid residue dissolved in (20 mL). The crude product was dialyzed at 4 °C and freeze dried afterwards yielding 52 mg of a dark red solid **23** (0.04 mmol, 97 %).

^1^H-NMR (700 MHz, DMSO-*d*_6_) δ [ppm] = 8.21 (bs, 1H, N*H*), 7.93 (s, 4H, H-2, H-5 Perylen, H-8, H-11 Perylen), 7.65 (dd, ^3^*J* = 21.2 Hz, ^4^*J* = 8.43 Hz, 8H, bay H-3, bay H-5), 7.42 (t, ^3^*J* = 7.6 Hz, 1H, peri H-4), 7.29 (d, ^3^*J* = 7.7 Hz , 2H, peri H-3, peri H-5), 6.97 (dd, ^3^*J* = 21.1 Hz, ^4^*J* = 8.5 Hz, 8H, bay H-2, bay H-6), 4.17- 4.02 (m, 2H, peri N-C*H*_2_-CH_2_), 3.68 (s, 2H, C*H*_2_-N_3_), 3.44 - 3.36 (m, 2H, peri N-CH_2_-C*H*_2_), 2.68 (sept, ^3^*J* = 6.7 Hz, 2H, peri C*H*-(CH_3_)_2_), 1.01 (d, ^3^*J* = 6.6 Hz, 12H, peri CH-(C*H*_3_)_2_; ^13^C-NMR (175 MHz, DMSO-*d*_6_) δ [ppm] = 168.1 (*C*ONH), 163.1 (bay C-1), 155.6 (C-1, C-6, C-7, C-12 Perylen), 146.0 (*C*ONCH_2_CH_2_), 145.0 (*C*ONAr), 132.9 (peri C-2, C-6), 132,5 (peri C-1), 131.0 (bay C-4), 129.8 (peri C‑4), 128.3 (bay C-3, C-5), 124.2 (peri C-3, C-5), 123.8 (C-3, C-4, C-9, C-10 Perylen), 123.0 (C_q_ Perylen), 120.5 (C-6a, C-6b, C-12a, C-12b Perylen), 120.0 (C-3a, C-9a Perylen), 119.6 (C-2, C-5, C-8, C-11 Perylen), 119.4 (bay C-2, C-6), 51.4 (*C*H_2_N_3_), 40.0 (CH_2_*C*H_2_NH), 37.0 (*C*H_2_CH_2_NH), 28.8 (*C*H(CH_3_)_3_), 24.2 (CH(*C*H_3_)_3_); HR-MS (ESI): m/z calculated for [C_64_H_48_N_6_O_21_S_4_]: 1365.35, found: 1364.18 MS (MALDI pos.): *m/z* (%) *=* 1387.08 (100) ([M+Na]^+^, ber. 1387.17), 1388.09 (92) ([M+Na]^+^, ber. 1388.08), 1403.06 (63) ([M+K]^+^, ber. 1403.28), 1404.04 (59) ([M+K]^+^, ber. 1404.28), 1409.05 (94) ([M+2Na]^+^, ber. 1410.07), 1410.03 (77) ([M+2Na]^+^, ber. 1411.07), UV-Vis (H_2_O): λ_max_(ε) = 563 (18073), 534 (15464), 450 (8203) nm; fluorescence (H_2_O, excitation 563 nm): λ_max_ = 617 nm; φ_f_ (H_2_O, RT, rhodamine B, 546 nm): 0.53.

## Spectra obtained for azide-modified perylene dye


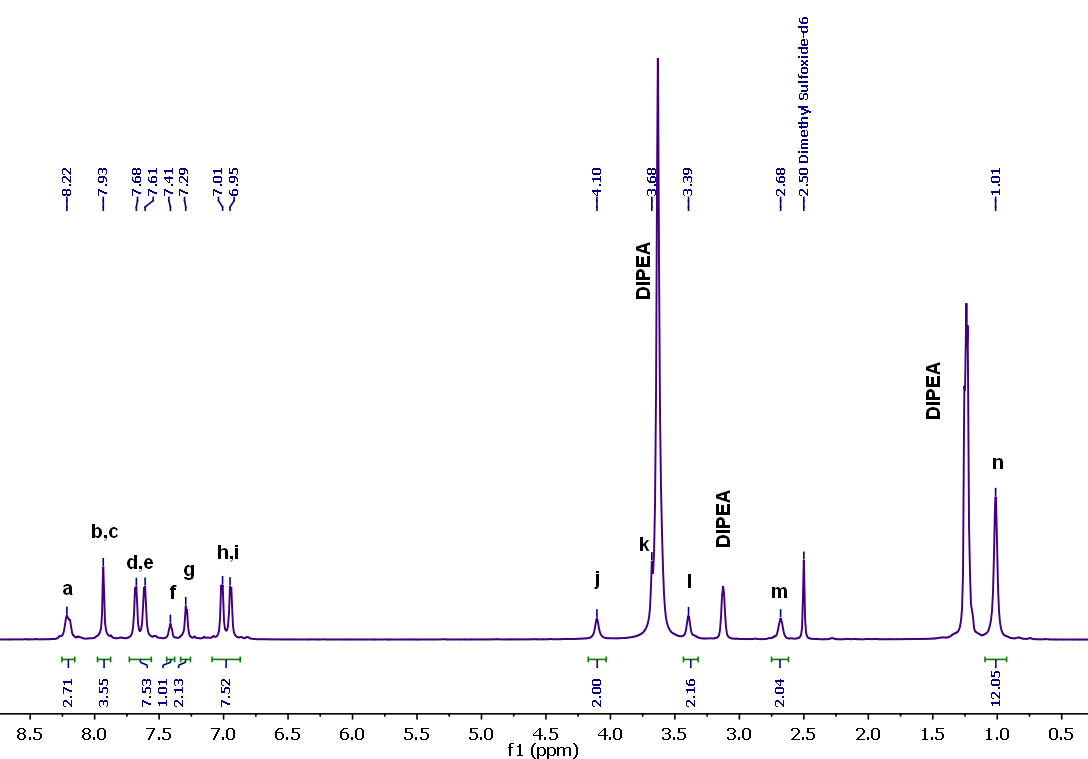


Supplementary Figure 14. ^1^H-NMR (700 MHz, DMSO-*d*_6_), compound 23.

# In addition to figure 2C:

Supplementary Table 1: *Statistical differences in IFN-α secretion by PBMCs after incubation with compounds 1-4*. Asterisks indicate the respective *P* values evaluated by ANOVA and Sidak’s multiple comparisons test; no declaration =  not significant (ns).

|  | 0.001 µg/mL | 0.01 µg/mL | 0.1 µg/mL | 1 µg/mL | 10 µg/mL |
| --- | --- | --- | --- | --- | --- |
| R848 (3) - RPA (4) | ns | * | *** | ns | *** |
| GQI (1) - GDA (2) | ns | ns | ns | *** | ns |
| GQI (1) - RPA (4) | ns | ns | ns | ns | ** |

# Cytotoxicity assay of commercial small molecules and azide-derivatives in PBMCs


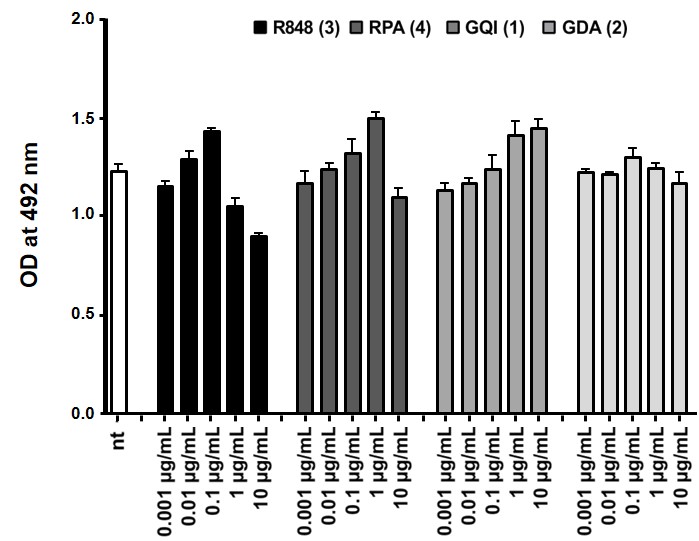


Supplementary Figure 15: *MTS assay of compounds 1-4 in PBMCs (nt = not treated).* Increasing concentrations of small molecules exhibit to some extend even a positive effect on cell viability compared to untreated PBMCs. Only for R848 the two highest and for RPA the highest concentration of 10 µg/mL show a minimal impact.

# RNA sequence information

**eGFP-mRNA:**

**G**GGAGACAAGCUUCCUGCAGGUCGACUCUAGAGGAUCCCGGGUACCGAGCUCGAAUUCGGCUUCCACC**AUG**GUGAGCAAGGGCGAGGAGCUGUUCACCGGGGUGGUGCCCAUCCUGGUCGAGCUGGACGGCGACGUAAACGGCCACAAGUUCAGCGUGUCCGGCGAGGGCGAGGGCGAUGCCACCUACGGCAAGCUGACCCUGAAGUUCAUCUGCACCACCGGCAAGCUGCCCGUGCCCUGGCCCACCCUCGUGACCACCCUGACCUACGGCGUGCAGUGCUUCAGCCGCUACCCCGACCACAUGAAGCAGCACGACUUCUUCAAGUCCGCCAUGCCCGAAGGCUACGUCCAGGAGCGCACCAUCUUCUUCAAGGACGACGGCAACUACAAGACCCGCGCCGAGGUGAAGUUCGAGGGCGACACCCUGGUGAACCGCAUCGAGCUGAAGGGCAUCGACUUCAAGGAGGACGGCAACAUCCUGGGGCACAAGCUGGAGUACAACUACAACAGCCACAACGUCUAUAUCAUGGCCGACAAGCAGAAGAACGGCAUCAAGGUGAACUUCAAGAUCCGCCACAACAUCGAGGACGGCAGCGUGCAGCUCGCCGACCACUACCAGCAGAACACCCCCAUCGGCGACGGCCCCGUGCUGCUGCCCGACAACCACUACCUGAGCACCCAGUCCGCCCUGAGCAAAGACCCCAACGAGAAGCGCGAUCACAUGGUCCUGCUGGAGUUCGUGACCGCCGCCGGGAUCACUCUCGGCAUGGACGAGCUGUACAAGUAAAGCGGCCGCGGAUCCCCGGGUACCGAGCU**AAAAAAAAAAAAAAAAAAAAAAAAAAAAAAAAAAAAAAAAAAAAAAAAAAAAAAAAAAAAAAAA**

**eGFP-siRNA strands:**

(All from IBA, Goettingen/Germany)

***MH533*** 5’-GAACUUCAGGGUCAGCUUGCCG-3’ (antisense)

***MH662*** 5’-GCAAGCUGACCCUGAAGUUC**X**U-3’ (sense)

**X**= C8-alkyne-dtCE phosphoramidite (Glen-Research)

***MH662-Gm8*** 5’-GCAAGCU**Gm**ACCCUGAAGUUCCU-3’ (sense)

# Tritium incorporation assay of 5’-mRNA capping reaction

Capping reactions for Cap0, Cap1 in stepwise- and one-step-format were pursued as described by the manufacturer with the *Vaccinia* Capping System and mRNA Cap 2’-*O*-methyltransferase (both from New England Biolabs, UK). The standard reaction volume was 70 µL for the methylation of 35 µg mRNA. As radioactive methyl-donor, 3 µL of [^3^H]-*S-*adenosyl methionine (^3^H-SAM, 1 µCi/µL, 80 Ci/ mmol, 12.5 µM, Hartmann Analytics, Germany) were used together with the SAM supplied with the enzymes. All reactions were monitored over 120 minutes, whereas for the stepwise Cap1-procedure, the 2‘-*O*-methyltransferase was added after 60 min.

Samples (8 µL) were spotted onto small Whatman filters (Roth) at indicated time points and precipitated in 5 % ice-cold trichloroacetic acid (TCA), followed by two washes at room temperature for 20 min and 10 min, respectively in 5 % TCA. The filters were swirled in ethanol, dried and transferred into scintillation vials including 3 mL of Ultima Gold MV liquid scintillation cocktail (PerkinElmer, Waltham, USA). Incorporated tritium was measured with a Wallac 1409 liquid scintillation counter (PerkinElmer, Waltham, USA) (20 sec/sample). The SAM-stock solution (1 µL) as a standard for specific activity.


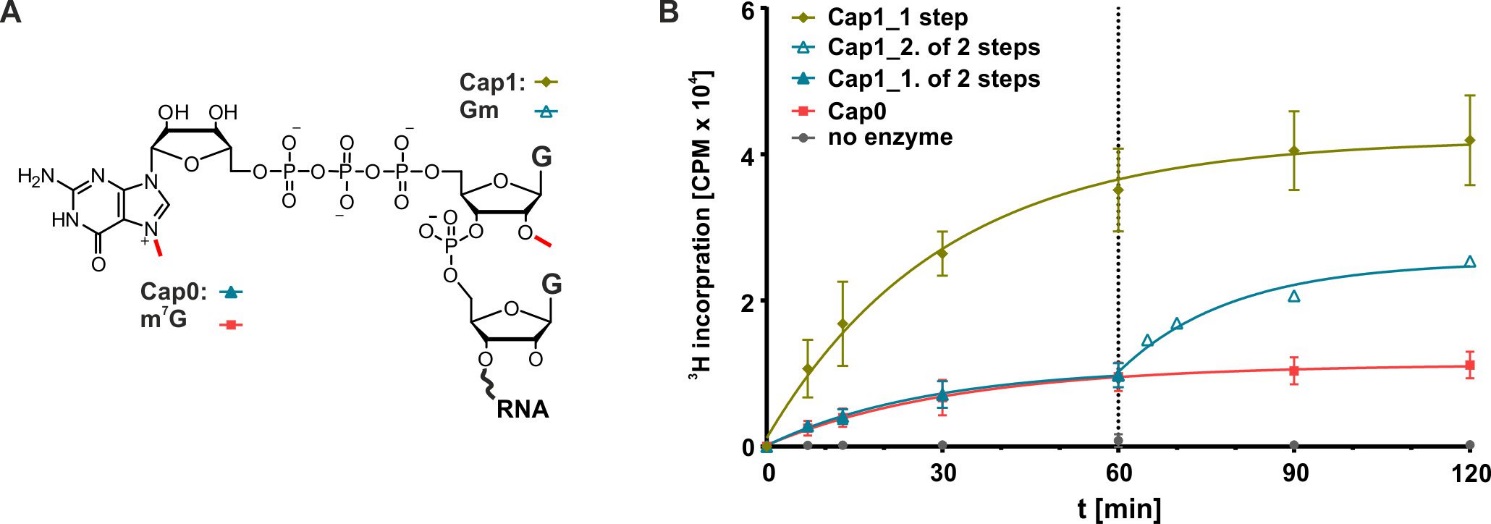


Supplementary Figure 16. *Investigation of capping reaction through tritium incorporation assay:* (A) Structures of Cap0 (m7G(5‘)pppG) and Cap1 (m7G(5‘)pppGm). (B) Methylation/Capping reactions of eGFP-mRNA monitored over 120 minutes for Cap0 only (orange), Cap1 in two steps (1. blue filled triangle, 2. blue framed triangle (one experiment only)) and in one step (green). A control reaction was carried out without enzymes. (n = 1-3; mean + SD).

# mRNA integrity after click-reaction

**
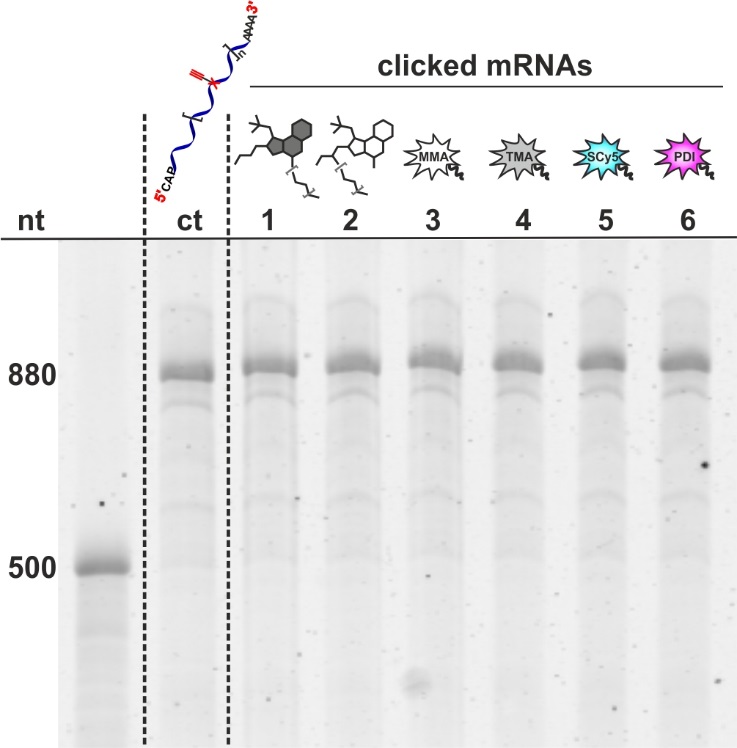
**

Supplementary Figure 17. *Exemplary degradation control after click-reaction of 10%-alkyne bearing eGFP-mRNA (6 % PAGE).* The gel was post-stained with Stains-all (Sigma-Aldrich), which was excited at 633 nm and emission signals were recorded at 670 nm on a Typhoon 9400 (GE Healthcare). First lane shows a random RNA (*in vitro* transcript) of 500 nucleotides (nt) for size comparison; ct (control) *in vitro* transcribed, untreated 10%-alkyne-mRNA; clicked with 1) RPA, 2) GDA, 3) MMA, 4) TMA, 5) SCy5 and 6) PDI. Clicked products show the same band shift as the untreated control, which confirms no impairment on RNA-stability after CuAAC reaction.

# Comparison of the effect of smTLRa, RNA and covalent conjugates of both in immunostimulation*.*


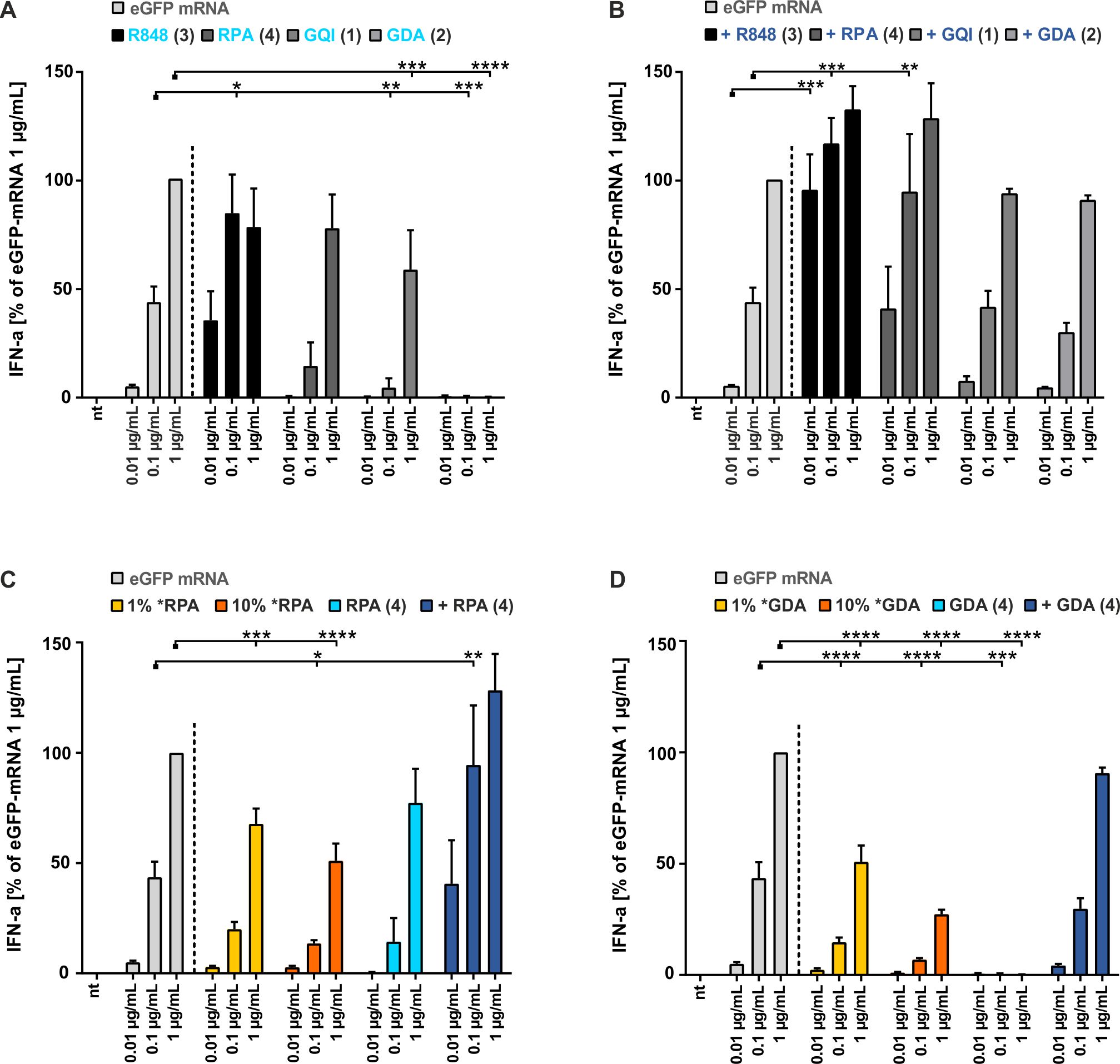


Supplementary Figure 18. *PBMC stimulation* (A) with eGFP-mRNA, commercial small molecules and their respective azide-derivatives (B) Fixed concentration of 0.1 µg/mL of SMs titrated individually with the indicated amounts of eGFP-mRNA. (C) Comparing results related to RPA. (D) Comparing results related to GDA. IFN-α production was measured by ELISA. Due to donor variation in the absolute amount of IFN-α secreted, data from each individual were normalized to 1.0 µg/mL eGFP-mRNA (= 100 %) (n = 3; mean + SD). (Asterisks above bars indicate the respective *P* values evaluated by ANOVA and Dunnett’s multiple comparisons test; no declaration = not significant (ns)).

# eGFP expression in human immature DCs


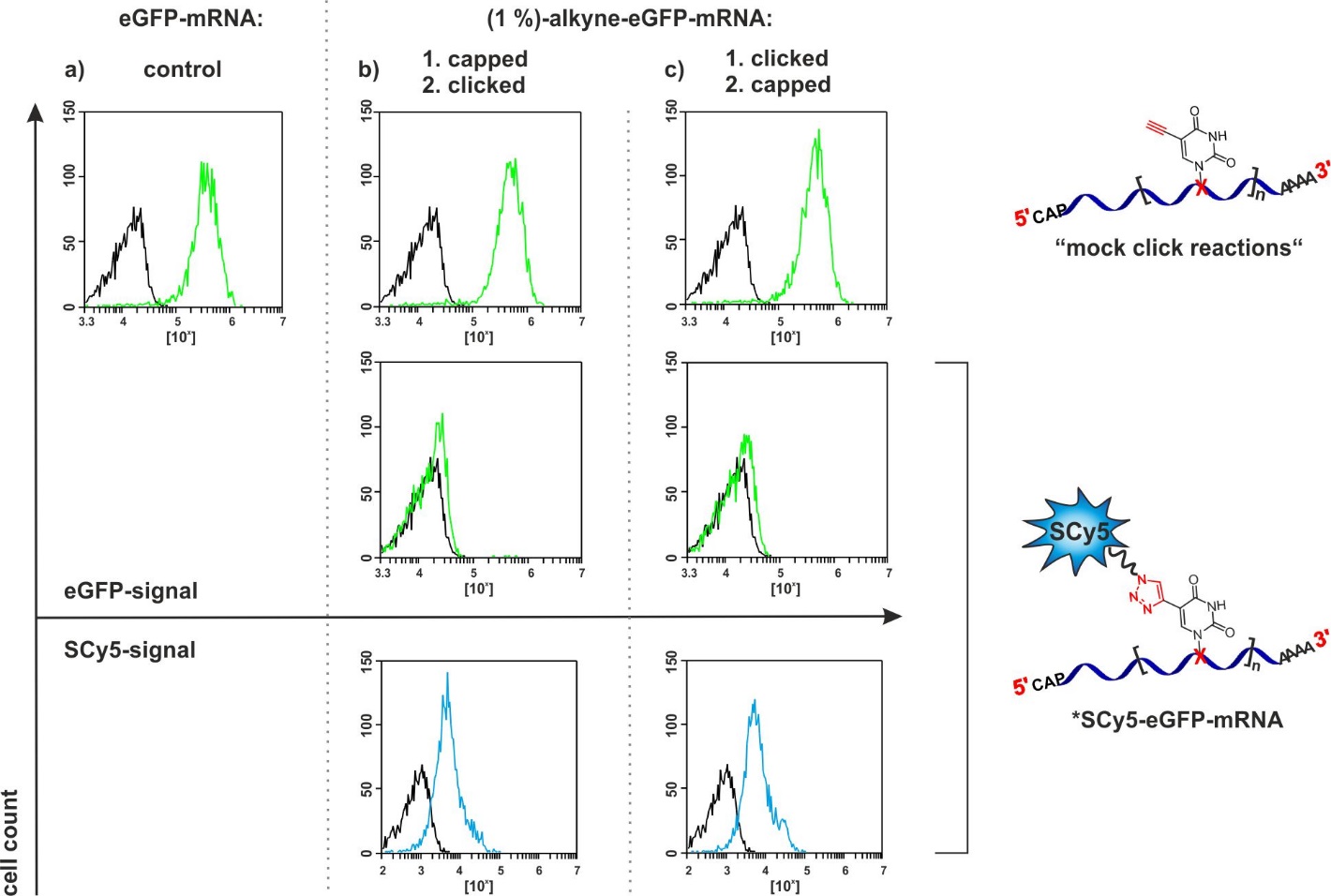


Supplementary Figure 19. *Electroporation of immature DCs:* a) Treatment of iDCs with capped unmodified eGFP-mRNA (green) and PBS (black) as control. Next, a (1 %)-alkyne-eGFP-mRNA was applied in two different ways: b) capping reaction first, followed by click reaction and c) *vice versa*. Mode b) and c) were both conducted in a “mock“-version, missing the azide-reaction partner (upper histograms) and with Sulfo-Cy5-azide as click-reaction partner (lower histograms including SCy5 signal in blue).

# References

1. Fulmer GR, Miller AJM, Sherden NH, Gottlieb HE, Nudelman A, Stoltz BM, Bercaw JE, Goldberg KI. NMR chemical shifts of trace impurities: Common laboratory solvents, organics, and gases in deuterated solvents relevant to the organometallic chemist. *Organometallics* (2010) **29**:2176–2179. doi:10.1021/om100106e

2. Percec V, Leowanawat P, Sun HJ, Kulikov O, Nusbaum CD, Tran TM, Bertin A, Wilson DA, Peterca M, Zhang S, et al. Modular synthesis of amphiphilic Janus glycodendrimers and their self-assembly into glycodendrimersomes and other complex architectures with bioactivity to biomedically relevant lectins. *J Am Chem Soc* (2013) **135**:9055–9077. doi:10.1021/ja403323y

3. Daly R, Vaz G, Davies AM, Senge MO, Scanlan EM. Synthesis and biological evaluation of a library of glycoporphyrin compounds. *Chem - A Eur J* (2012) **18**:14671–14679. doi:10.1002/chem.201202064

4. Morales CP, Catalán J, Domingo V, González Delgado JA, Dobado JA, Herrador MM, Quílez Del Moral JF, Barrero AF. Protecting-group-free synthesis of chokols. *J Org Chem* (2011) **76**:2494–2501. doi:10.1021/jo102280n

5. Zhao J, Wei S, Ma X, Shao H. A mild and environmentally benign method for the synthesis of glycals in PEG-600/H2O. *Green Chem* (2009) **11**:1124. doi:10.1039/b821681a

6. Lill A, Scholich K, Stark H. Synthesis of novel dansyl-labeled Celecoxib derivatives. *Tetrahedron Lett* (2013) **54**:6682–6686. doi:10.1016/j.tetlet.2013.09.025

7. Bakleh ME, Sol V, Estieu-Gionnet K, Granet R, Déléris G, Krausz P. An efficient route to VEGF-like peptide porphyrin conjugates via microwave-assisted “click-chemistry.” *Tetrahedron* (2009) **65**:7385–7392. doi:10.1016/j.tet.2009.07.028

8. Peneva K, Mihov G, Nolde F, Rocha S, Hotta JI, Braeckmans K, Hofkens J, Uji-i H, Herrmann A, Müllen K. Water-soluble monofunctional perylene and terrylene dyes: Powerful labels for single-enzyme tracking. *Angew Chemie - Int Ed* (2008) **47**:3372–3375. doi:10.1002/anie.200705409
